# Two years and counting: a prospective cohort study on the scope and severity of post-COVID symptoms across diverse patient groups in the Netherlands—insights from the CORFU study

**DOI:** 10.1136/bmjopen-2024-093639

**Published:** 2025-09-11

**Authors:** Dorthe O Klein, Sophie F Waardenburg, Emma B N J Janssen, Marieke S J N Wintjens, Maike Imkamp, Stella C M Heemskerk, Erwin Birnie, Gouke J Bonsel, Michiel C Warlé, Lotte M C Jacobs, Bea Hemmen, Jeanine Verbunt, Bas C T van Bussel, Susanne van Santen, Bas L J H Kietelaer, Gwyneth Jansen, Frederikus A Klok, Martijn D de Kruif, Kevin Vernooy, Juanita A Haagsma, Folkert W Asselbergs, Marijke Linschoten, Jochen W L Cals, Hugo Ten Cate, Iwan C C van der Horst, Nick Wilmes, CAPACITY-COVID Collaborative Consortium, AK Al-Ali, Chahinda Ghossein-Doha, Sander M J van Kuijk

**Affiliations:** 1 Department of Clinical Epidemiology and Medical Technology Assessment, Maastricht University Medical Centre+, Maastricht, The Netherlands; 2 Care and Public Health Research Institute (CAPHRI) Maastricht University, Maastricht, The Netherlands; 3 Department of Anesthesiology and Pain Medicine, Maastricht University Medical Centre+, Maastricht, The Netherlands; 4 Department of Obstetrics and Gynaecology, Maastricht University Medical Centre+, Maastricht, The Netherlands; 5 Cardiovascular Research Institute Maastricht (CARIM) Maastricht University, Maastricht, The Netherlands; 6 Department of Intensive Care Medicine, Maastricht University Medical Centre+, Maastricht, The Netherlands; 7 Department of Public Health, Erasmus University Medical Centre, Rotterdam, The Netherlands; 8 EuroQol Research Foundation, Rotterdam, The Netherlands; 9 Department of Surgery, Radboud University Medical Centre, Nijmegen, The Netherlands; 10 Rehabilitation Medicine, Functioning, Participation & Rehabilitation, Maastricht University, Maastricht, The Netherlands; 11 Adelante Centre of Expertise in Rehabilitation and Audiology, Hoensbroek, The Netherlands; 12 Cardiovascular Disease, Mayo Clinic, Rochester, Minnesota, USA; 13 Department of Cardiology, Zuyderland Medical Centre, Heerlen, The Netherlands; 14 Department of Thrombosis and Hemostasis, Leiden University Medical Centre, Leiden, The Netherlands; 15 Department of Pulmonology, Zuyderland Medical Centre, Heerlen, The Netherlands; 16 Department of Cardiology, Maastricht University Medical Centre+, Maastricht, The Netherlands; 17 Department of Cardiology, Amsterdam University Medical Centres, Amsterdam, The Netherlands; 18 The National Institute for Health Research University College London Hospitals Biomedical Research Centre, London, UK; 19 Institute of Health Informatics, University College London, London, UK; 20 Netherlands Heart Institute, Utrecht, The Netherlands; 21 Department of Family Medicine, Maastricht University, Maastricht, The Netherlands; 22 Department of Biochemistry, Maastricht University Medical Centre+, Maastricht, The Netherlands; 23 Thrombosis Expert Centre Maastricht, Maastricht University Medical Centre+, Maastricht, The Netherlands; 24 Department of Internal Medicine, Maastricht University Medical Centre+, Maastricht, The Netherlands; 25 Department of Cardiology, Erasmus University Medical Centre, Rotterdam, The Netherlands

**Keywords:** Post-Acute COVID-19 Syndrome, Prevalence, Patients, COVID-19

## Abstract

**Importance:**

Little research has been done on post-COVID symptoms at 24 months postinfection and on the association these may have on health-related quality of life (HRQOL).

**Objective:**

We assessed the prevalence and severity of post-COVID symptoms and quantified EuroQol 5 Dimension 5 Level (EQ-5D-5L), self-perceived health question (EuroQol Visual Analogue Scale (EQ-VAS)) and health utility scores (HUS) up to 24 months follow-up.

**Design:**

The longitudinal multiple cohort CORona Follow-Up (CORFU) study combines seven COVID-19 patient cohorts and a survey among the general public. The participants received questionnaires on several time points. Participants were stratified by: without a known SARS-CoV-2 infection (control group), proven SARS-CoV-2 infection but non-hospitalised, proven SARS-CoV-2 infection hospitalised to the ward, and proven SARS-CoV-2 infection hospitalised to the intensive care unit (ICU).

**Setting:**

In this study, data of seven COVID-19 patient cohorts and a survey among the general public are included.

**Participants:**

Former COVID-19 patients and controls participated in this cohort study.

**Main outcomes and measures:**

Former COVID-19 patients and non-COVID-19 controls were sent questionnaires on symptoms associated with post-COVID condition. The CORFU questionnaire included 14 symptom questions on post-COVID condition using a five-level Likert-scale format. Furthermore, HRQOL was quantified using the EuroQol EQ-5D-5L questionnaire: EQ-VAS and the EQ-5D-5L utility score. The EQ-5D-5L questionnaire includes five domains that are scored on a five-point Likert scale: mobility, self-care, usual activities, pain/discomfort and anxiety/depression.

**Results:**

A total of 901 participants (and 434 controls) responded at 24 months follow-up. In all former COVID-19 patients, the presence of post-COVID condition at 24 months was observed in 62 (42.5%, 95% CI 34.3% to 50.9%) of the non-hospitalised patients, 333 (65.0%, 95% CI 60.7% to 69.2%) of the hospitalised ward patients and 156 (63.2%, 95% CI 56.8% to 69.2%) of the ICU patients, respectively (p<0.001). The most common symptoms included fatigue, sleep problems, muscle weakness/pain and breathing issues, with hospitalised participants reporting most often having symptoms. Multiple post-COVID symptoms were significantly associated with EQ-5D-5L measures. The mean and SD of the EQ-VAS were 71.6 (17.9), 70.0 (17.3) and 71.4 (17.5) for non-hospitalised, ward and ICU participants, respectively, and 75.6 (17.7) for the controls (p<0.001). The HUS resulted in 0.81 (0.20), 0.77 (0.19) and 0.79 (0.22) for non-hospitalised, hospitalised ward and ICU participants, respectively, and 0.84 (0.19) for the control group (CG) (p<0.001).

**Conclusions:**

Many former COVID-19 patients experience post-COVID symptoms at 24 months follow-up, with the highest prevalence in hospitalised participants. Also, former patients reported a lower HRQOL.

**Trial registration number:**

The CORFU study was registered at clinicaltrials.gov (registration number NCT05240742).

**Strengths and limitations of this study**

- The long follow-up period up until 24 months after infection with the SARS-CoV-2 virus allows for estimating long-term post-COVID symptom prevalence and severity.
- By grouping several cohorts, we were able to include a diverse population of patients and controls that enabled us to provide information on diverse groups based on sex, body mass index, age and severity of disease.
- The control population helps to shed light on the prevalence and severity of the same symptoms that are used to diagnose post-COVID condition.
- Selection bias cannot be ruled out, as specific subgroups of former COVID-19 patients may not have participated in CORona follow-up at equal rates.
- It is possible that the control group contained more cases of (asymptomatic) COVID-19 patients than identified, as people may have contracted COVID-19 without noticing. This could have resulted in some misclassification of controls.

## Introduction

 Post-COVID condition refers to a range of symptoms that persist or that begin at least 3 months after the acute phase of the COVID-19 disease. The prevalence of post-COVID condition differs widely in the literature, although it fluctuates around 50% of the former hospitalised patients who still experience symptoms even 12 months after the acute SARS-CoV-2 infection.^1 2^ Although over 200 different symptoms have been identified to be associated with post-COVID condition, the majority of symptoms are rare.^3^ Most prevalent symptoms include difficulties with cognition (eg, memory loss and brain fog), physical impairment (eg, postexertional symptom exacerbation, malaise and muscle/joint pain), gastrointestinal discomfort (eg, abdominal pain and nausea), respiratory symptoms (eg, pain when breathing and coughing) and cardiovascular symptoms (eg, palpitations, postural orthostatic tachycardia syndrome and swollen ankles or feet).^3 4^

The long-lasting symptoms can have a major impact on the patient’s daily life, especially when they are severe.^5 6^ The severity of the impact of these symptoms can be measured by assessing the health-related quality of life (HRQOL).^7 8^ Post-COVID condition may also impede regular participation in society, such as with social activities or work.^3 7 9^ These symptoms have been shown to have an important psychological impact on the lives of patients and their relatives.^10^

Several studies have shown that the severity of the acute illness is associated with the presence and severity of persistent symptoms in post-COVID condition up to 1 year after infection.^11^,^14^ Additionally, the presence of pre-existing chronic diseases, comorbidities and a history of hospitalisation for COVID-19 have been identified as risk factors for the development of post-COVID condition, further emphasising the association between disease severity and long-term complications.^3 15^ Our study aim was to assess the (excess) prevalence and severity of post-COVID symptoms from 3 months up to 2 years after SARS-CoV-2 infection and compare symptoms to controls without known SARS-CoV-2 infection in the past. We hypothesised that the prevalence is strongly dependent on disease severity during the acute COVID-19 phase even 2 years after infection, and that, although symptoms are not unique to post-COVID condition, a substantial excess disease burden would be seen compared with controls. A secondary aim was to determine self-perceived health and HRQOL of patients with post-COVID condition at 24 months.

## Methods

### Design and study population

**Figure 1:**
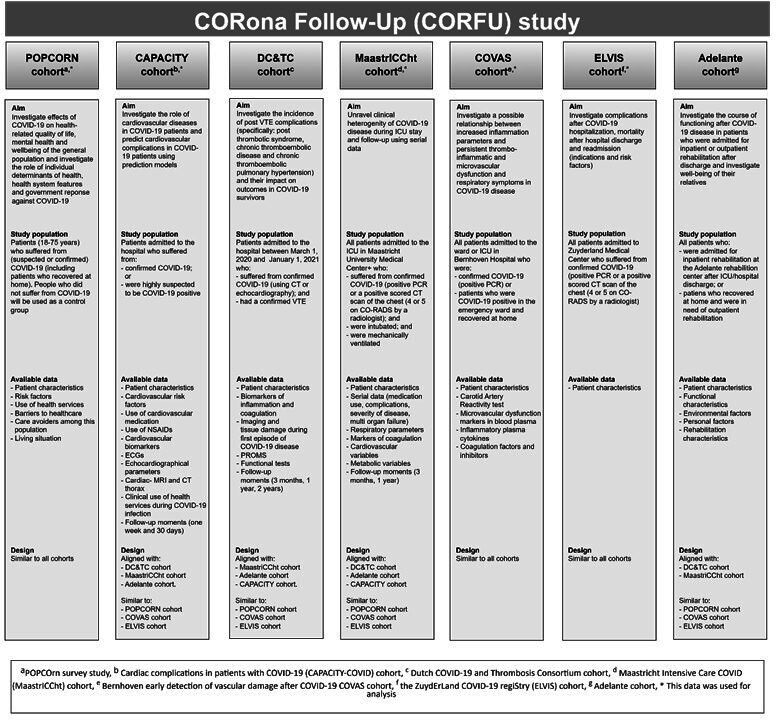
Overview of cohorts in the CORFU study. CORFU, CORona Follow-Up; ICU, intensive care unit; POPCOrn, POPulation health impact of the COVID-19 pandemic.

The longitudinal multiple cohort CORona Follow-Up (CORFU) study combines data from seven Dutch COVID-19 patient cohorts (figure 1) and a self-report survey among the general public, as extensively reported in the study design. Patient and public involvement is also described in this protocol article.^16^ Data from the following cohorts were combined: the Maastricht Intensive Care COVID cohort,^17 18^ the Bernhoven early detection of vascular damage after COVID-19 cohort cohort,^19^ the ZuydErLand COVID-19 regiStry cohort^20^ and the cardiac complications in patients with COVID-19 cohort^21^ and the community-based POPulation health impact of the COVID-19 pandemic (POPCORN) cohort.^22 23^ The latter cohort predominantly consisted of controls without a known SARS-CoV-2 infection and was subsequently regarded as non-COVID controls. POPCORN participants who reported to have suffered from (mild) COVID-19 were counted as cases. The participants of POPCORN were recruited by an international market research agency that distributed and launched the questionnaire. The participants were members of the market research agency’s existing voluntary panels. Participants in all cohorts had to be at least 18 years of age, and SARS-CoV-2 cases were either confirmed by PCR or CT scan (COVID-19 Reporting and Data System with a score of 4–5) or were suspected cases, as there was limited testing capacity at the start of the pandemic. All participants of the included studies were considered eligible and were asked to complete one or more questionnaires after consent. A waiver was obtained from the medical research ethics committee of Maastricht University Medical Centre+ and Maastricht University (METC 2021–2990) and METCs of the participating cohorts.^16^ The CORFU study was registered at clinicaltrials.gov (registration number NCT05240742).

### CORFU questionnaire

Former COVID-19 patients and non-COVID-19 controls were sent one or more questionnaires (depending on the cohort) on symptoms associated with post-COVID condition. CORFU participants were divided into four subgroups: (1) participants without a known SARS-CoV-2 infection, that is, the controls, (2) participants with proven or suspected SARS-CoV-2 infection without hospitalisation, that is, non-hospitalised, (3) participants with proven SARS-CoV-2 infection with hospital ward admission, that is, general ward and (4) participants with SARS-CoV-2 infection with intensive care unit (ICU) admission. Participants of the cohorts were invited to complete the CORFU questionnaire at 3, 6, 12, 18 and 24 months after initial SARS-CoV-2 infection. Participants were contacted by email and could fill in the questionnaire via a web-based survey or, if requested, on paper.

The timing of the CORFU questionnaire was determined by the participant’s date of first infection (diagnosis and/or admission). Due to the timing of study initiation, some participants could not complete earlier questionnaires, depending on the cohort; most participants completed either one or two during follow-up. Depending on the cohort and patient preference, questionnaires were completed either digitally or on paper.

**Figure 2:**
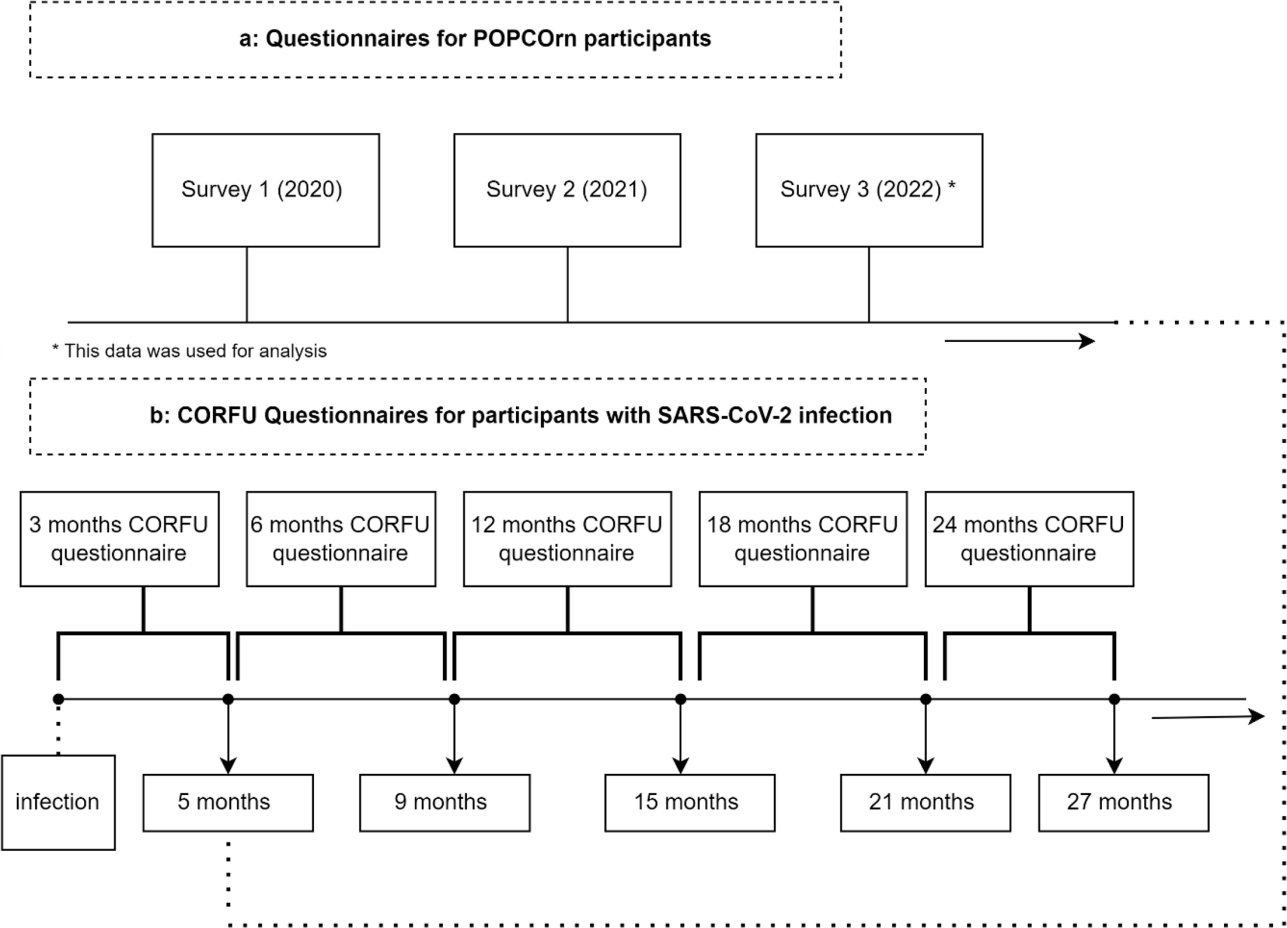
Questionnaires. CORFU, CORona Follow-Up; POPCORN, POPulation health impact of the COVID-19 pandemic.

Survey participants of the POPCORN study received separate questionnaires at set calendar times between 22 April 2020 and 26 June 2022, approximately 1 year apart. For the non-COVID control group, we used data from the third survey, as those questionnaires were completed closest in calendar time to the period CORFU questionnaires were sent to former patients (figure 2). Data were collected between 1 October 2021 and 31 December 2023.

### Outcome variables

The primary outcome was the prevalence and severity of post-COVID symptoms at 24 months after initial infection. CORFU participants were defined as having post-COVID condition if at least one symptom was present 3 months after initial infection and was not pre-existent. Pre-existing symptoms present before the SARS-CoV-2 infection were only regarded as post-COVID symptoms if there was deterioration after infection. Secondary outcomes were the severity of post-COVID symptoms at other follow-up moments and HRQOL.

### Data collection

The CORFU questionnaire included 14 symptom questions on post-COVID condition using a five-level Likert severity scale format (range: ‘not present’ to ‘extremely severe’). The symptoms included fatigue, headache, dizziness, muscle weakness or muscle pain, coughing, dyspnoea, pain when breathing, angina pectoris, palpitations, cognitive problems, loss of smell or taste, sleep problems, loss of appetite and swollen ankles or feet.^22 24^ Headache as a symptom was added to the CORFU questionnaire after 28 June 2022, as early studies reported headache as a symptom associated with post-COVID condition.^14^ Furthermore, HRQOL was quantified using the EuroQol 5 Dimension 5 Level (EQ-5D-5L) questionnaire: self-perceived health question (EuroQol Visual Analogue Scale (EQ-VAS)) and the EQ-5D-5L utility score based on the Dutch tariff. *This Dutch value set was applied to the responses to calculate a utility score, which is anchored on a scale where 1 represents the ‘full health’ and 0 represents the ‘death’.*^25^ The EQ-5D-5L questionnaire includes five domains that are scored on a five-point Likert scale: mobility, self-care, usual activities, pain/discomfort and anxiety/depression. The EQ-5D-5L utility score is calculated as a weighted sum of the score of the responses using a value set (scale 0–1), which reflects societal preferences for EQ-5D-5L health states.^26^ The EQ-VAS (a part of EQ-5D-5L) is a self-rated visual analogue scale assessing an individual’s perceived health state, ranging from 0 (‘the worst imaginable health state’) to 100 (‘the best imaginable health state’). In addition, we asked for vaccination status (having had one or more vs none).

Education has been used as an indicator for socioeconomic status.^27^ We have categorised the level of education into basic, intermediate and advanced, as suggested by the suggestion of the International Standard Classification of Education (ISCED).^28^

### Statistical analysis

The source population consisted of participants of the previously developed cohort studies. Characteristics of CORFU participants at baseline (ie, at SARS-CoV-2 diagnosis or when receiving the first survey questionnaire) were expressed as mean and SD for continuous variables, and count and percentage for categorical variables. Characteristics at baseline were stratified by subgroup (non-hospitalised, ward, ICU and control) and tested using Pearson’s χ^2^ test or one-way analysis of variance.

First, we separately visualised the proportion of responses to the five-level symptoms questions using floating stacked bar charts for the four subgroups at all follow-up moments. All bivariate correlations between symptoms at 24 months were computed using Spearman’s correlation coefficients and visualised using a correlation plot.

Second, we dichotomised symptoms associated with post-COVID condition into being present or absent. A symptom was registered as ‘present’ when it was scored at least moderately severe (three or above on a five-point Likert scale). Symptom prevalence was presented as count and percentage, and we used multivariable logistic regression analysis to test differences between subgroups adjusted for age and sex. Next, based on the presence of at least one non-pre-existing post-COVID symptom or deterioration of a pre-existing symptom, we categorised the three subgroups of cases into having any post-COVID symptom or none. We computed the percentage of patients vaccinated at the time of completing their 24-month questionnaire and computed the percentage of vaccinated and unvaccinated patients that exhibited post-COVID symptoms, stratified by the four subgroups.

EQ-5D domain scores, EQ-VAS and the EQ-5D-5L utility scores were stratified by subgroup and tested using multivariable linear regression analysis. We adjusted for age, sex and comorbidities based on a directed acyclic graph. Univariable and multivariable linear mixed-effects regression analysis on data from all follow-up moments was used to estimate the association between post-COVID symptoms separately and combined, and the EQ-VAS and between post-COVID symptoms and the HRQOL utility score.

Analyses were performed using R V.4.0.2 (The R Foundation for Statistical Computing, Vienna University of Economics and Business, Vienna, Austria). The p values of 0.05 or lower were considered to indicate statistical significance and 95% CIs were computed if appropriate.

### Patient and public involvement

Patient organisations (Family and Patient-Centred Intensive Care (FCIC), IC Connect and the ‘Hartenraad’) and patients of the Maastricht University Medical Centre+ (MUMC+) Intensive Care panel were involved in the design of the CORFU study. Patients were involved in the development and testing of the international basic questionnaire on persistent symptoms after COVID-19, which serves as the basis for the CORFU questionnaire. In addition, patients provided feedback on the phrasing of questions, the fill-out time of the questionnaire and the willingness to fill out the questionnaire periodically. Participants will be able to provide feedback on the (missing) content of the CORFU questionnaire through an open-ended question. Comments will be discussed and implemented prospectively when deemed relevant, making the CORFU questionnaire a continuously developing measurement instrument. Patients will have an advisory role in developing the patient platform prototype (WP4), which allows patients to digitally consult their answers in real time and compare them with reference populations. In addition, advice will be asked on the (type of) feedback questions provided, the formatting and visualisation of answers and the relevant reference groups to be considered. Eventually, CORFU findings will be presented in a lay summary, and a flyer on long COVID will be developed in close collaboration with patients. The dissemination strategy of CORFU findings and the long COVID flyer will be based on patient and public preferences, in which also the involved patient organisations will have an important role.

## Results

We included 4291 participants who completed a total of 5523 questionnaires (table 1). This included 3086 (72.0%) non-COVID controls. Over all follow-up moments after infection, we included 266 (6.2%) non-hospitalised patients, 581 (13.5%) former ward patients and 358 (8.3%) former ICU patients. We received the most questionnaires from former COVID-19 patients on the 24-month follow-up moment, that is, 904.

**Table 1:** Number of completed questionnaires for every time point presented for each subgroup of participants

	Controls without COVID-19 (n=3086)	Non-hospitalised COVID-19 patients (n=266)	Hospitalised COVID-19 patients (ward) (n=581)	Hospitalised COVID-19 patients (ICU) (n=358)
Survey 1, April–May 2020*	3086	203	5	2
Survey 2, May–June 2021*	372	71	1	0
Survey 3, April–May 2022	434	0	0	0
3 months after initial COVID-19 infection		49	1	3
6 months after initial COVID-19 infection		51	5	30
12 months after COVID-19		61	19	54
18 months after COVID-19		57	48	70
24 months after COVID-19		146	511	247

* Participants of the survey study (ie, POPCORN) who reported having had COVID-19 at home or were admitted to hospital ward or ICU were post hoc classified as non-hospitalised, ward or ICU patients, respectively, and hence, contributed questionnaires to those groups. Note that column totals equal more that the number of participants per group, as participants may have completed questionnaires at multiple time points.

ICU, intensive care unit; POPCORN, POPulation health impact of the COVID-19 pandemic.

The mean age was lowest in the control group, with 47.7 years compared with the non-hospitalised (52.3 years), hospitalised general ward (64.9 years) and ICU patients (62.0 years). The male-to-female ratio was close to equal in the control group (48.7% male), but in the clinical subgroups, substantially more men than women were present, with the highest percentage of men in the ICU subgroup (72.9%). In the control group and the non-hospitalised patients subgroup, asthma was most often reported as a chronic disease (11.5% and 10.9%, respectively). For the ward patients, this was diabetes and arrhythmia or palpitations, followed closely by osteoarthritis and low back pain. In the ICU patients, diabetes and osteoarthritis were most often reported as a comorbidity, followed by arrhythmia or palpitations (table 2).

**Table 2:** Characteristics of study participants stratified by subgroup

	Controls without COVID-19 (n=3086)	Non-hospitalised COVID-19 patients (n=266)*	Hospitalised COVID-19 patients (ward) (n=581)	Hospitalised COVID-19 patients (ICU) (n=358)	Total number of patients (4.291)	P value for difference
Sex (male), n (%)	1501 (48.7%)	121 (45.5%)	360 (62.0%)	261 (72.9%)	2243 (52.3%)	<0.001
Age (yrs), mean (SD)	47.7 (16.8)	52.3 (13.9)	64.9 (11.6)	62.0 (10.1)	51.5 (16.9)	<0.001
BMI (kg/m^2^), mean (SD)	N.A.	27.4 (4.1)	27.9 (5.2)	29.1 (5.1)	28.3 (5.1)	0.002
Education,† n (%)						<0.001
Basic	406 (13.2%)	51 (19.2%)	145 (25.5%)	84 (23.7%)	686 (16.0%)	
Intermediate	1308 (42.4%)	108 (40.6%)	272 (47.8%)	162 (45.8%)	1850 (43.3%)	
Advanced	1372 (44.5%)	107 (40.2%)	152 (26.7%)	108 (30.5%)	1739 (40.7%)	
Comorbidities,‡ n (%)						
Arrhythmia/ palpitations	8 (1.8%)	17 (6.4%)	97 (16.7%)	48 (13.4%)	170 (10.4%)	<0.001
Asthma	355 (11.5%)	27 (10.2%)	71 (12.2%)	38 (10.6%)	491 (11.4%)	0.792
Chronic bronchitis	13 (3.0%)	12 (4.5%)	86 (6.5%)	17 (4.8%)	80 (4.9%)	0.077
DM type 1 or 2	297 (9.6%)	21 (7.9%)	97 (16.7%)	56 (15.6%)	471 (11.0%)	<0.001
Lung emphysema	12 (2.8%)	3 (1.1%)	24 (4.1%)	11 (3.1%)	50 (3.1%)	0.112
Angina pectoris	4 (0.9%)	10 (3.8%)	29 (5.0%)	21 (5.6%)	63 (3.8%)	<0.001
Heart failure	13 (3.0%)	5 (1.9%)	27 (4.7%)	10 (2.8%)	55 (3.4%)	0.149
Prior stroke or CVA	69 (2.2%)	9 (3.4%)	31 (5.3%)	16 (4.5%)	125 (2.9%)	<0.001
Hernia or severe back pain	268 (8.7%)	26 (9.8%)	85 (14.6%)	31 (8.7%)	410 (9.6%)	<0.001
Osteoarthritis	195 (6.3%)	19 (7.1%)	83 (14.3%)	54 (15.1%)	351 (8.2%)	<0.001
Prior knee or hip replacement	10 (2.3%)	6 (2.3%)	38 (6.5%)	15 (4.2%)	69 (4.2%)	<0.001
Chronic rheumatoid arthritis	166 (5.4%)	12 (4.5%)	32 (5.5%)	13 (3.6%)	223 (5.2%)	0.507
Prior or current malignancy	87 (2.8%)	7 (2.6%)	31 (5.3%)	21 (5.9%)	146 (3.4%)	<0.001
Living situation, n (%)						<0.001
Alone	817 (26.5%)	58 (21.8%)	117 (20.2%)	52 (14.7%)	1043 (24.4%)	
With parents	184 (6.0%)	8 (3.0%)	0 (0.0%)	2 (0.6%)	194 (4.5%)	
With partner without child(ren)	1063 (34.4%)	102 (38.3%)	341 (58.9%)	213 (60.2%)	1718 (40.1%)	
With partner and child(ren)	663 (21.5%)	67 (25.2%)	93 (16.1%)	67 (18.9%)	890 (20.8%)	
Alone with child(ren)	193 (6.3%)	24 (9.0%)	17 (2.9%)	15 (4.2%)	249 (5.8%)	
Other	166 (5.4%)	7 (2.6%)	11 (1.9%)	5 (1.4%)	189 (4.4%)	

* 72% of non-hospitalised participants were derived from the control survey, and hence, data not available in the control survey affect availability of data in non-hospitalised patients.

† Education: low (ie, primary education or lower secondary education), medium (ie, upper secondary education or postsecondary non-tertiary education) and high (first or second stage of tertiary education) based on ISCED classification.

‡ Not all cohorts contributed complete data on these variables.

BMI, body mass index; CVA, cerebral vascular accident; DM, diabetes mellitus; ICU, intensive care unit; ISCED, International Standard Classification of Education; n, number of participants; N.A., not assessed.

### Prevalence and severity of post-COVID symptoms

**Figure 3:**
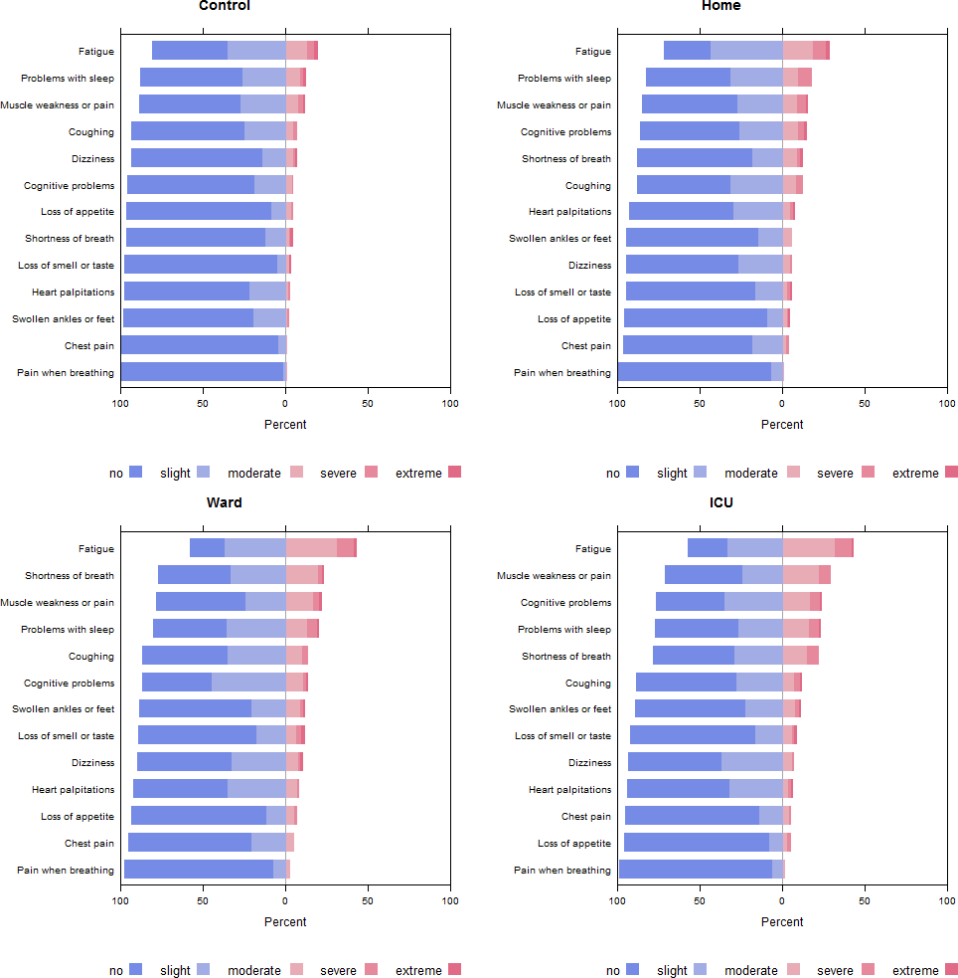
Distribution of five-point Likert scores at 24 months stratified by subgroup. Note that the symptoms are ordered from most to least prevalent, and this may differ between subgroups. Data of participants who completed the third survey and had not contracted COVID-19 by then were used, as those questionnaires were completed closest in time to the calendar period of the 24 months questionnaires, that is, between April and May 2022. ICU, intensive care unit.

All 14 symptoms from the CORFU questionnaire were found in the four subgroups at 24 months (see table 3). These differed significantly between the four subgroups, except for loss of appetite. Fatigue had the highest prevalence in all subgroups of patients but was highest in the former hospitalised patients. The most prevalent symptoms reported in the control group were fatigue (19.1%), sleep problems (12.0%) and muscle weakness or pain (11.3%). For non-hospitalised patients, the most prevalent symptoms were fatigue (28.8%), sleep problems (17.8%) and muscle weakness/pain (15.8%). The most prevalent symptoms in ward patients were fatigue (45.4%), shortness of breath (25.4%) and muscle weakness (25.0%). In former ICU patients, these were fatigue (42.5%), muscle weakness or pain (28.7%) and cognitive problems (24.1%) (figure 3).

**Table 3:** Prevalence of post-COVID symptoms stratified by subgroup at 24 months after initial infection

Symptom	Controls without COVID-19 (n=434)*	Non-hospitalised COVID-19 patients (n=146)	Hospitalised COVID-19 patients (ward) (n=511)	Hospitalised COVID-19 patients (ICU) (n=247)	P value for difference†
Fatigue	83 (19.1%)	42 (28.8%)	232 (45.4%)	105 (42.5%)	<0.001
Headache‡	30 (6.9%)	11 (12.4%)	19 (13.5%)	3 (6.4%)	0.022
Dizziness	11 (2.5%)	9 (6.2%)	56 (11.1%)	16 (6.5%)	<0.001
Muscle weakness/ pain	49 (11.3%)	23 (15.8%)	127 (25.0%)	71 (28.7%)	<0.001
Coughing	30 (6.9%)	18 (12.3%)	75 (14.9%)	26 (10.5%)	0.050
Shortness of breath	17 (3.9%)	18 (12.3%)	129 (25.4%)	55 (22.3%)	<0.001
Pain when breathing	3 (0.7%)	2 (1.4%)	15 (3.0%)	2 (0.8%)	0.023
Chest pain	3 (0.7%)	5 (3.4%)	29 (5.7%)	11 (4.5%)	0.007
Heart palpitations	7 (1.6%)	11 (7.1%)	45 (8.8%)	17 (6.9%)	0.002
Cognitive problems	18 (4.1%)	21 (14.4%)	81 (15.9%)	59 (24.1%)	<0.001
Loss of smell or taste	12 (2.8%)	9 (6.2%)	66 (12.9%)	21 (8.5%)	<0.001
Problems with sleep	52 (12.0%)	26 (17.8%)	113 (22.1%)	55 (22.3%)	<0.001
Loss of appetite	17 (3.9%)	7 (4.8%)	31 (6.1%)	9 (3.6%)	0.628
Swollen ankles or feet	28 (6.5%)	9 (6.2%)	72 (14.2%)	28 (11.4%)	0.019

* Data of participants who completed the 2022 survey and had not contracted COVID-19 by then were used, as those questionnaires were completed closest in time to the calendar period of the 24 months questionnaires.

† Adjusted for age, sex and comorbidities.

‡ Headache was not available in all questionnaires, and hence, denominators may differ from those of other symptoms.

ICU, intensive care unit.

In all former COVID-19 patients, the presence of post-COVID condition at 24 months was observed in 62 (42.5%, 95% CI 34.3% to 50.9%) of the non-hospitalised patients, 333 (65.0%, 95% CI 60.7% to 69.2%) of the hospitalised ward patients and 156 (63.2%, 95% CI 56.8% to 69.2%) of the ICU patients, respectively (p<0.001). The percentage of former patients that reported having two or more symptoms was 21.9%, 44.3% and 41.7% for non-hospitalised, ward and ICU patients, respectively. The number of former patients that reported having three or more symptoms was 14.4%, 32.0% and 24.7% (both p<0.001). The proportion of responses to the five-level symptom questions at different timepoints is shown in online supplemental figures S1-S4.

Of all former COVID-19 patients, 89.0% (95% CI 81.2% to 94.4%) had been vaccinated at least once at 24 months. We observed large differences in vaccination rates across subgroups. The lowest rates were found in the non-hospitalised patient group (67.1%, 95% CI 58.9% to 74.7%) and the highest in the patients admitted to the ward (95.7%, 95% CI 93.6% to 97.3%). In the former ICU patients, this was 87.9% (95% CI 83.1% to 91.7%, p value for difference between groups<0.001). At 24 months, 43.9% (95% CI 33.9% to 54.3%) of vaccinated non-hospitalised patients had at least one post-COVID symptom present compared with 39.6% (95% CI 25.8% to 54.7%) of non-vaccinated non-hospitalised patients (p=0.753), 65.3% (95% CI 60.9 to 69.5) of vaccinated patients admitted to the ward had at least one symptom compared with 59.1% (95% CI 36.4 to 79.3) for non-vaccinated patients (p=0.712) and 64.1% (95% CI 57.3 to 70.4) of ICU patients had at least one symptom present compared with 56.7% (95% CI 37.4 to 74.5) for non-vaccinated ICU patients (p=0.559).

Online supplemental figure S5 shows positive correlations between symptoms for all four subgroups between post-COVID symptoms at 24 months after initial infection. However, these correlations were weak (around or below Spearman’s r of 0.5), except for the correlation between shortness of breath and fatigue (Spearman’s r of 0.55).

### Post-COVID symptoms and HRQOL

At 24 months after infection, mean self-rated health on the EQ-VAS was 75.6 (95% CI 73.9 to 77.2) for the control group, 71.6 (95% CI 68.7 to 74.5) for non-hospitalised patients and 70.0 (95% CI 68.5 to 71.5) and 71.4 (95% CI 69.2 to 73.6) for participants admitted to the ward or ICU, respectively. The mean EQ-VAS of the control group was significantly higher than that of the combined former COVID-19 patients (mean difference: 4.9, 95% CI 2.9 to 6.9, p<0.001). This pattern was comparable for the EQ-5D-5L utility score: mean scores were 0.84 (95% CI 0.83 to 0.86), 0.81 (95% CI 0.78 to 0.84), 0.77 (95% CI 0.76 to 0.79) and 0.79 (95% CI 0.76 to 0.82) for the control group, non-hospitalised patients, patients admitted to the ward and patients admitted to the ICU, respectively. The mean difference between controls and former COVID-19 patients was 0.06 in favour of controls (95% CI 0.04 to 0.08, p<0.001).

Patients with post-COVID condition had a mean EQ-VAS score of 64.4 (95% CI 62.9 to 65.8), which was significantly lower than those who had COVID-19 but no post-COVID condition (mean of 80.4, 95% CI 79.1 to 81.7). The mean difference was 16.0 (95% CI 14.1 to 18.0, p<0.001). The mean utility score for patients with post-COVID condition was 0.71 (95% CI 0.69 to 0.73), again lower than those without post-COVID condition (mean utility: 0.90, 95% CI 0.89 to 0.91). The mean difference was 0.19 (95% CI 0.17 to 0.21, p<0.001).

**Figure 4:**
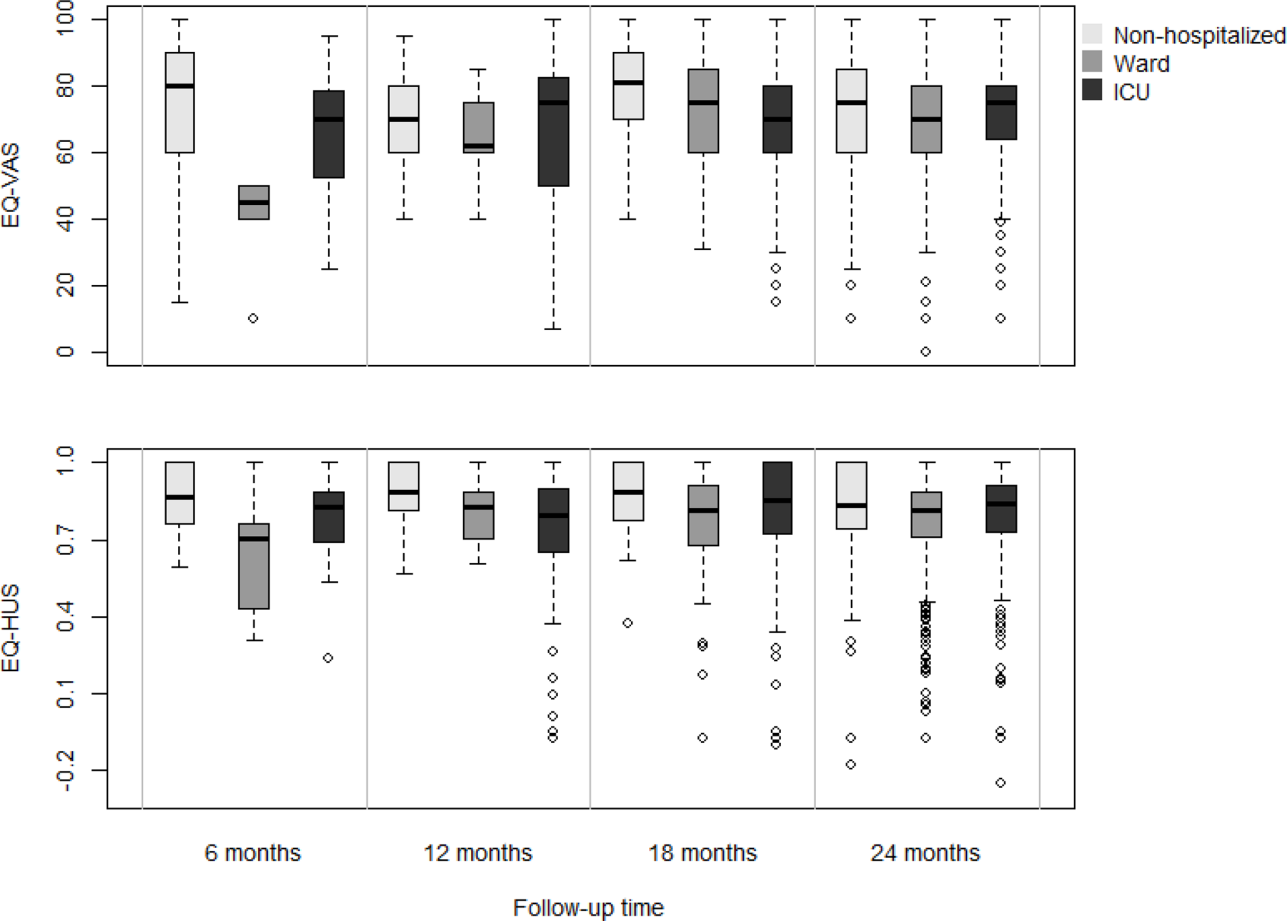
HRQOL expressed as the EQ-5D-5L VAS score and the EQ-HUS, for former COVID-19 patients stratified by follow-up time. A vertical line for patients admitted to the ward at 18 months is due to the fact that too few questionnaires were available to estimate the distribution. Note that no data of controls are presented as all presented data are relative to the index date of infection with SARS-CoV-2. EQ-5D-5L, EuroQol 5 Dimension 5 Level; HRQOL, health-related quality of life; HUS, health utility score; ICU, intensive care unit; VAS, Visual Analogue Scale.

Boxplots of HRQOL of former COVID-19 patients stratified by follow-up moment are shown in figure 4. Table 4 shows that after adjustment for other symptoms, fatigue, muscle weakness or pain, problems with cognition, shortness of breath, loss of appetite and swollen ankles or feet were significantly associated with self-rated health on the EQ-VAS. Similar associations, except for muscle weakness or pain and swollen ankles or feet, were seen with the EQ-5D-5L utility score (online supplemental table ST1).

**Table 4:** Associations between post-COVID symptoms and the EQ-VAS score in former COVID-19 patients

	Univariable		Multivariable	
	Regression coefficient (95% CI)	P value	Regression coefficient (95% CI)	P value
Fatigue	−11.0 (−11.8 to −10.2)	<0.001	−7.5 (−8.5 to −6.4)	<0.001
Headache*	−8.3 (−10.2 to −6.3)	<0.001	−0.4 (−3.1 to 2.4)	0.710
Dizziness	−0.1 (−0.3 to 0.1)	0.358	0.0 (−0.1 to 0.2)	0.780
Muscle weakness or pain	−0.6 (−0.8 to −0.3)	<0.001	−0.4 (−0.6 to −0.1)	0.009
Coughing	−5.9 (−7.1 to −4.7)	<0.001	−0.6 (−1.6 to 0.4)	0.214
Shortness of breath	−9.6 (−10.6 to −8.7)	<0.001	−3.1 (−4.2 to −2.0)	<0.001
Pain with breathing	−0.2 (−0.4 to 0.1)	0.171	−0.1 (−0.3 to 0.0)	0.133
Chest pain	−0.4 (−0.6 to 0.1)	0.006	0.1 (−0.2 to 0.5)	0.446
Heart palpitations	−0.3 (−0.5 to −0.1)	0.002	−0.2 (−0.4 to 0.1)	0.221
Cognitive problems	−8.5 (−9.5 to −7.4)	<0.001	−2.2 (−3.2 to −1.1)	<0.001
Loss of smell or taste	−0.2 (−0.5 to 0.2)	0.338	0.2 (0.0 to 0.5)	0.087
Problems with sleep	−6.4 (−7.4 to −5.4)	<0.001	−0.6 (−1.5 to 0.3)	0.156
Loss of appetite	−9.9 (−11.5 to −8.3)	<0.001	−3.3 (−4.7 to −1.9)	<0.001
Swollen ankles or feet	−0.4 (−0.8 to −0.1)	0.017	0.5 (0.1 to 0.9)	0.025

* Headache was not available in all questionnaires. Hence, univariable and multivariable analysis, including headache, was performed on available cases. The multivariable analysis included all symptoms.

EQ-VAS, EuroQol Visual Analogue Scale.

## Discussion

In this study, more than half of all former hospitalised COVID-19 patients were classified as having post-COVID condition 24 months after initial infection. In former non-hospitalised patients, this was two out of five patients.

At 24 months, fatigue, sleep problems, muscle weakness or muscle pain were most prevalent symptoms in all patient subgroups and the control group. Fatigue was most often observed in this study, which is in line with previous findings,^7 9 29^ and this appears to be related to disease severity.^7 30 31^ In the hospitalised patients (ward and ICU), shortness of breath also had a higher prevalence at 24 months. Furthermore, cognitive dysfunction was more prevalent in ICU patient subgroups. These last findings are in line with a previous study that showed that cognitive impairment was higher in the most severely ill COVID-19 patients.^32^

We hypothesised that the severity of the acute disease was a predictor for the presence of post-COVID symptoms at 24 months, but this was only valid for the symptoms cognitive dysfunction and severity of fatigue. However, postintensive care syndrome (PICS) could also have played a role in this in the ICU-admitted severity group. However, we have no means to discriminate PICS from post-COVID, as its presentation can be so much alike.

Symptoms 24 months after acute infection between the former ward and ICU patients differed only slightly, although treatment in the ICU differed (patients with SARS-CoV-2 were sedated and mechanically ventilated during their ICU admission).

Although we expected that the EQ-5D-5L and VAS results would be lower at 24 months due to the severity during the acute phase for former ICU patients compared with patients who had not been admitted to the hospital, this was not the case in our study. The results indicate that the severity of the acute SARS-CoV-2 infection alone does not predict HRQOL in the long term. It may be possible that the domains measured with the EQ-5D-5L were not affected much by the post-COVID symptoms. Therefore, we recommend future psychometric research to assess the sensitivity of the EQ-5D-5L questionnaire and the long-term complaints of post-COVID condition. At the same time, our study population consisted mostly of patients with an age above 50 years. This could mean that these patients already had lower EQ-5D-5L scores before initial infection. Unfortunately, this information was unavailable. However, our study population seems representative since our findings are comparable wih international studies.^33^

Another explanation could be that former hospital patients were earlier admitted to a more intensive rehabilitation programme compared with the non-admitted patients. This is also seen in the POPCORN cohort and by healthcare use (rehabilitation and physiotherapy) in this group. Thus, it may be that this group recovers faster compared with the non-admitted patients.^34^

In the current study, mean EQ-VAS and EQ-5D-5L utility scores were higher than described by Gerritzen *et al.*^34^ This may have been due to different timeliness, as their study had a shorter follow-up period of 14 months postinfection. Also, their study population differed, as only a small proportion of hospitalised COVID-19 patients were included. This specific population was identified as one with high healthcare demands, which may explain the low scores on the EQ-VAS and utility score.^34^ The average EQ-VAS score reported by Huang *et al*^7^ in China, assessed at 24 months postinfection, reached a mean score of 80 for former COVID-19 patients and 85 for matched non-COVID-19 controls. This was higher than the scores found in our study for all three groups of former COVID patients and controls. Moreover, the average utility score of 1 found in this Chinese study for former COVID patients only applied to those with a full- or part-time job prior to infection. The reason for these differences may be in part due to cultural differences between the Netherlands and China. Our study could make a comparison between former COVID-19 patients and controls. Both the EQ-VAS and utility score were higher in the latter group, representing better health in the control group. Taken together, it appears that even, after 2 years of recovering from a SARS-CoV-2 infection, the ongoing health impact on former patients remains present. This also includes possible delay and/or reduced capacity in work reintegration and participation in daily activities, such as household and social activities.^35^

Over the last 2 years, different health organisations have provided various definitions of post-COVID condition, varying specifically in onset and persistence of the long-lasting symptoms that identify post-COVID condition. One of the most often used definitions was proposed by the WHO in April 2023 in a consensus document based on a Delphi process.^36^ While consensus was reached regarding the duration and timing of the symptoms, there is no consensus on which specific symptoms constitute post-COVID condition and the severity level at which these symptoms should persist. Likely, post-COVID condition is a very heterogeneous disease, and therefore, clustering homogeneous groups may help narrow down the clinically relevant differences in patients with post-COVID condition. This may engage the conversation about applying cut-off scores for different clusters in the number and severity of symptoms. Recent literature has identified various possible risk factors for developing post-COVID condition, such as female sex, older age, smoking and severity of infection. These risk factors may contribute to further improving the definition and prediction of post-COVID condition.^3^

Regarding vaccination status, patients admitted to the hospital had the highest and non-hospitalised patients with the lowest vaccination rate. Since most of the patients in our study population had a SARS-CoV-2 infection before the vaccination rounds started, we could not determine any causal relation between vaccination status and long-term symptoms.

### Strengths and limitations

This study has several strengths. First, the long follow-up period up until 24 months after infection with the SARS-CoV-2 virus allows for estimating long-term post-COVID symptom prevalence and severity. Also, by grouping several cohorts and data from a survey, we were able to include a diverse population of patients and controls that enabled us to provide information on diverse groups based on sex, body mass index, age and severity of disease. The control population helps to shed light on the prevalence and severity of the same symptoms that are used to diagnose post-COVID condition. However, the control group was slightly younger on average, more often highly educated, and had fewer chronic diseases. Our study also has several limitations. We cannot rule out selection bias as specific subgroups of former COVID-19 patients may not have participated in CORFU at equal rates. For instance, patients who did not experience any burden in daily life or perceive any symptoms, or those with such severe post-COVID symptoms that completing questionnaires poses too much of a burden, may have refrained from responding. This could have resulted in underestimating the number of patients with severe complaints. Also, in our study, the number of hospitalised patients was overrepresented compared with those who stayed at home during the acute phase of the infection. To adjust for this, we showed the stratified results, and within every subgroup, we presented the prevalence of post-COVID condition within every subgroup. Furthermore, it is possible that the control group contained cases of unidentified COVID-19 patients. *At the time the control participants were recruited, there was only very limited availability of COVID-19 tests.* This could have resulted in some misclassification of controls. Finally, we did not have vaccination status relative to completing each follow-up questionnaire, making it impossible to distinguish any potential vaccination effect on symptoms associated with post-COVID condition.

## Conclusion

In conclusion, many former COVID-19 patients still experience one or more post-COVID symptoms up to 2 years after initial infection, with the highest prevalence in former hospitalised patients. The most common symptoms observed in all former COVID-19 groups included fatigue, sleep problems, muscle weakness or pain and breathing issues, with fatigue being notably the most common symptom. Furthermore, HRQOL of former patients was comparable 2 years after their infection, regardless of the severity of the initial disease. This emphasises the need for further investigation of the underlying mechanisms and treatment options for former COVID-19 patients with post-COVID symptoms. In addition, healthcare services (including rehabilitation) may be needed to support this large group of former COVID-19 patients.

## Supplementary material

10.1136/bmjopen-2024-093639 online supplemental file 1

10.1136/bmjopen-2024-093639 online supplemental file 2

## Data Availability

Data are available on reasonable request.
