# Supplementary material for: Two years and counting: a prospective cohort study on the scope and severity of post-COVID symptoms across diverse patient groups in the Netherlands—insights from the CORFU study
Source: BMJ Open. 2025 Sep 11;15(9):e093639. doi: 10.1136/bmjopen-2024-093639 (PMC12519329; doi:10.1136/bmjopen-2024-093639)
Supplement: online supplemental file 1 [file bmjopen-15-9-s001.docx]

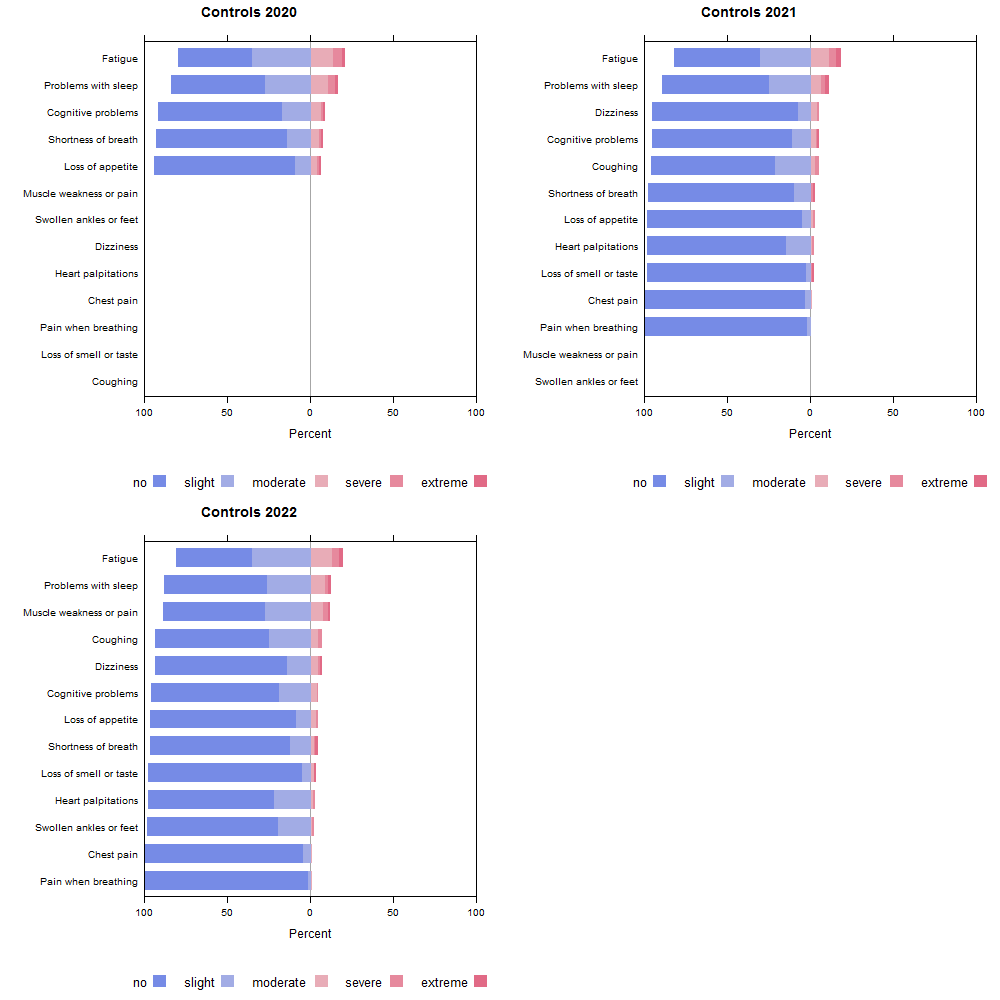


*Supplementary figure S1. Distribution of 5-point Likert scores for the three surveys taken in 2020, 2021, and 2022. Over time, more symptoms associated with post-COVID condition were added. Symptoms are ordered from most to least prevalent. Symptoms that remain blank were not measured that year.*


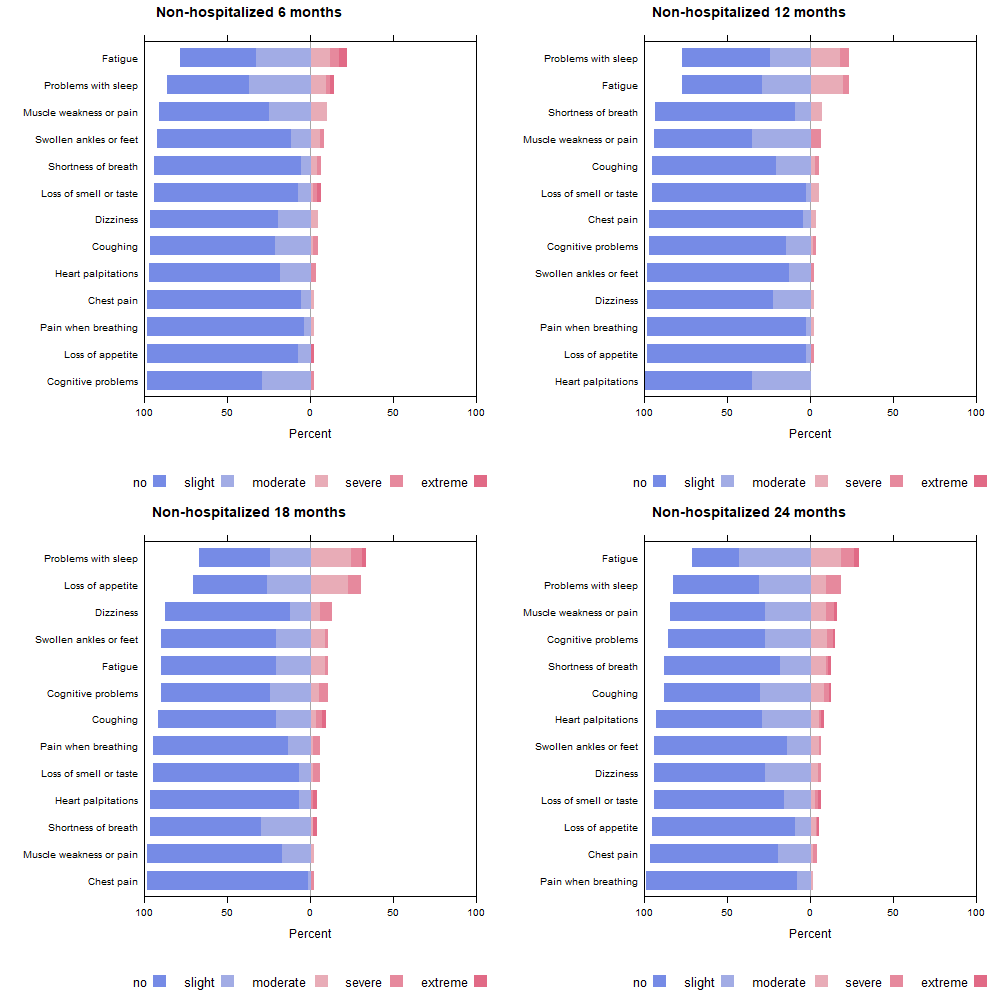


*Supplementary figure S2. Distribution of 5-point Likert scores for non-hospitalized patients over time. Symptoms are ordered from most to least prevalent.*


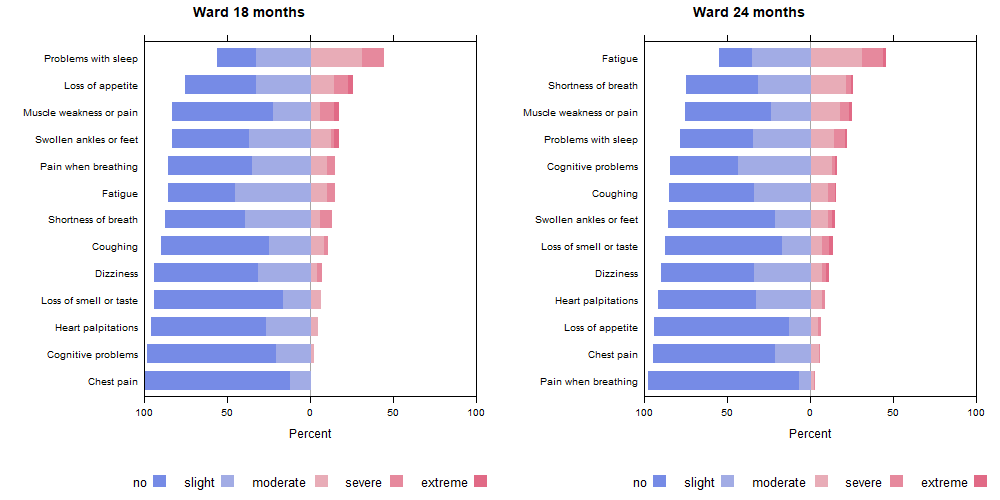


*Supplementary figure S3. Distribution of 5-point Likert scores for patients admitted to the ward over time. At 6 and 12 months, too few observations were available. Symptoms are ordered from most to least prevalent.*


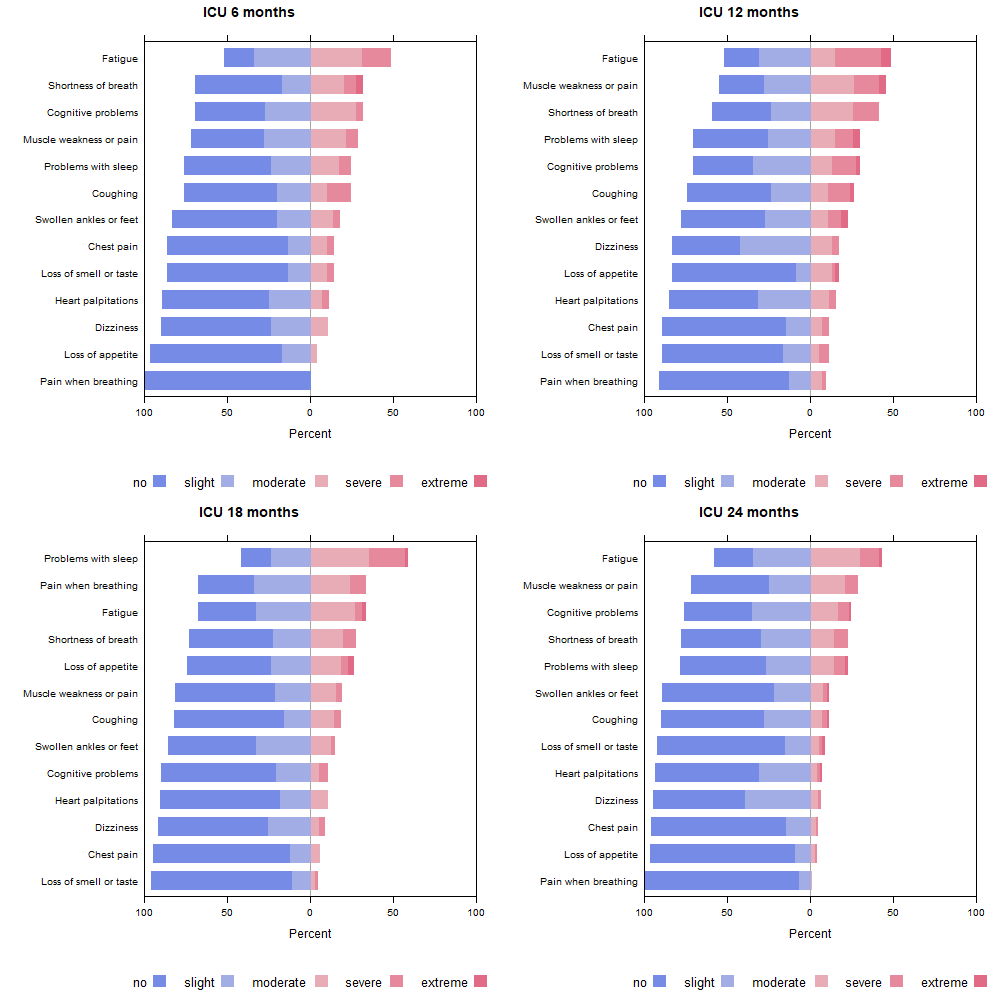


*Supplementary figure S4. Distribution of 5-point Likert scores for patients admitted to the ICU over time. Symptoms are ordered from most to least prevalent.*


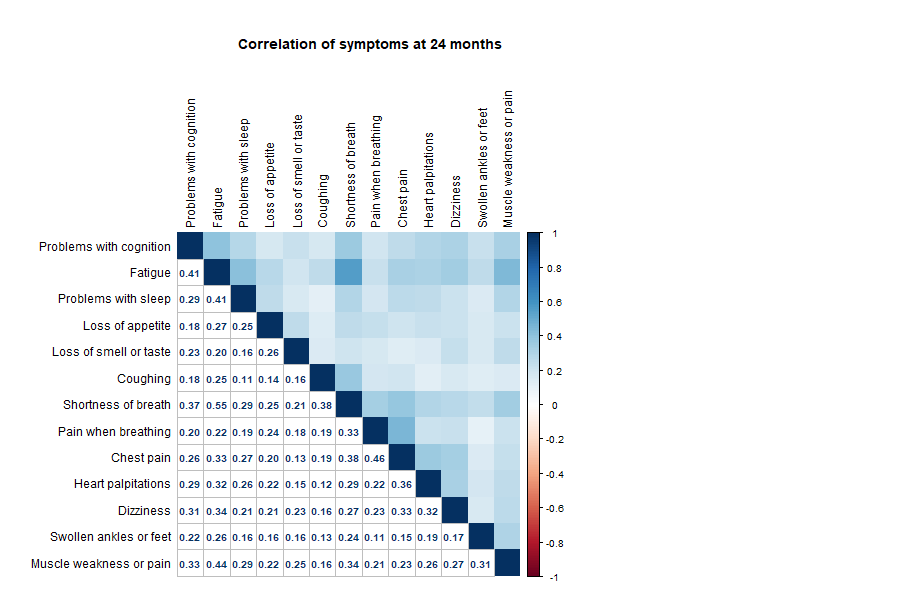


*Supplementary figure 5. Bivariate correlations between post-COVID symptoms at 24 months after initial infection, expressed as Spearman’s rho.*
