# Supplementary material for: Two years and counting: a prospective cohort study on the scope and severity of post-COVID symptoms across diverse patient groups in the Netherlands—insights from the CORFU study
Source: BMJ Open. 2025 Sep 11;15(9):e093639. doi: 10.1136/bmjopen-2024-093639 (PMC12519329; doi:10.1136/bmjopen-2024-093639)
Supplement: online supplemental file 2 [file bmjopen-15-9-s002.docx]

*Supplementary table 1. Associations between post-COVID symptoms and health-related quality of life utility score in former COVID-19 patients^*^*

|  | **Univariable** | | **Multivariable** | |
| --- | --- | --- | --- | --- |
|  | **Regression coefficient (95% CI)** | **P-value** | **Regression coefficient (95% CI)** | **P-value** |
| Fatigue | -0.116 (-0.126; -0.107) | <0.001 | -0.061 (-0.073; -0.049) | <0.001 |
| Headache^‡^ | -0.117 (-0.141; -0.094) | <0.001 | -0.012 (-0.042; 0.019) | 0.310 |
| Dizziness | -0.001 (-0.003; 0.001) | 0.227 | 0.000 (-0.002; 0.001) | 0.744 |
| Muscle weakness or pain | -0.004 (-0.007; -0.002) | <0.001 | -0.001 (-0.003; 0.001) | 0.272 |
| Coughing | -0.066 (-0.079; -0.052) | <0.001 | -0.004 (-0.016; 0.008) | 0.506 |
| Shortness of breath | -0.109 (-0.120; -0.097) | <0.001 | -0.037 (-0.049; -0.024) | <0.001 |
| Pain with breathing | -0.001 (-0.004; 0.002) | 0.506 | 0.000 (-0.003; 0.002) | 0.811 |
| Chest pain | -0.005 (-0.008; -0.002) | 0.002 | 0.001 (-0.003; 0.005) | 0.663 |
| Heart palpitations | -0.004 (-0.006; -0.001) | 0.004 | 0.001 (-0.002; 0.004) | 0.704 |
| Cognitive problems | -0.109 (-0.121; -0.096) | <0.001 | -0.043 (-0.055; -0.030) | <0.001 |
| Loss of smell or taste | -0.006 (-0.010; -0.002) | 0.005 | -0.001 (-0.005; 0.002) | 0.370 |
| Problems with sleep | -0.080 (-0.092; -0.069) | <0.001 | -0.018 (-0.029; -0.007) | 0.002 |
| Loss of appetite | -0.128 (-0.147; -0.110) | <0.001 | -0.054 (-0.071; -0.038) | <0.001 |
| Swollen ankles or feet | -0.008 (-0.012; -0.004) | <0.001 | -0.002 (-0.006; 0.003) | 0.488 |

*^‡^Headache was not available in all questionnaires. Hence, univariable and multivariable analysis including headache was performed on available cases. The multivariable analysis included all symptoms.*

*^*^Health-related quality of life utility score was measured using the EQ-5D-5L utility score (ranging from -0.4 to 1)*
