# Supplementary material for: Two years and counting: a prospective cohort study on the scope and severity of post-COVID symptoms across diverse patient groups in the Netherlands—insights from the CORFU study
Source: BMJ Open. 2025 Sep 11;15(9):e093639. doi: 10.1136/bmjopen-2024-093639 (PMC12519329; doi:10.1136/bmjopen-2024-093639)
Supplement: Supplementary file 3 [file bmjopen-2024-093639.reviewer_comments.pdf]

PEER REVIEW HISTORY

BMJ Open publishes all reviews undertaken for accepted manuscripts. Reviewers are asked to complete a checklist review form and are provided with free text boxes to elaborate on their assessment. These free text comments are reproduced below.

ARTICLE DETAILS

Title (Provisional)

Two Years and Counting: A Prospective Cohort Study on the Scope and Severity of Post-COVID Symptoms Across Diverse Patient Groups in the Netherlands – Insights from the CORFU Study

Authors

Klein, Dorthe; Waardenburg, Sophie; Janssen, Emma BNJ; Wintjens, Marieke SJN; Imkamp, Maike; Heemskerk, Stella CM; Birnie, Erwin; Bonsel, Gouke J.; Warle, Michiel; Jacobs, Lotte MC; Hemmen, Bea; Verbunt, Jeanine; van Bussel, Bas C.T.; van Santen, Susanne; Kietelaer, Bas; Jansen, Gwyneth; Klok, Frederikus A; de Kruif, Martijn D.; Vernooy, Kevin; Haagsma, Juanita; Asselbergs, Folkert W; Linschoten, Marijke; Cals, Jochen WL; ten Cate, Hugo; van der Horst, Iwan C.C.; Wilmes, Nick; consortium , CAPACITY-COVID; Ghossein-Doha, Chahinda; van Kuijk, Sander

---

VERSION 1 - REVIEW

**Reviewer**	**1**
**Name**	**Greenwood, Darren**
**Affiliation**	**University of Leeds, School of Medicine**
**Date**	**30-Sep-2024**
**COI**	**I am co-investigator on a number of service evaluations and longitudinal cohorts investigating the long-term experience of persistent symptoms in people living with long covid.**

General comments

1. This is a hugely important topic. A large and growing number of people have now been living with long covid for over two years. Reported quality of life is measurable worse than many better understood chronic conditions, but with relatively little research into what to do about it. The impact on those individuals lives is substantial. The impact on reduced workforce and increased benefit payments is also significant.

2. This paper offers the potential to track persistence of symptoms in the same cohort over 2 years, though follow-up is poor and repeated measures few.

Major comments

3. The study benefits from pooling rich individual participant data from seven longitudinal cohort studies, but the total sample size remains quite small for a prevalence study across subgroups, presumably because of the longitudinal nature of the data collection and subsequent losses to follow-up. Initial response rates have not been presented, only the numbers included at each time point. Losses to follow-up are quantified and shown to be around 90%, leaving substantial room for bias. There is strong potential for people with persistent symptoms to be more motivated to respond, or less able to respond, and for this to be different between the groups.

4. The study benefits from a healthy control sample missing from many similar studies. The participants appears to be dominated by the two larger cohorts (CAPACITY-COVID and POPCOrn), with controls appearing to come just from POPCOrn, but hospitalised and ICU patients from the other cohorts. The sampling methods of the controls is unclear in both the manuscript and the protocol paper referenced. The reference within the protocol points to a manuscript describing recruitment by an international market research agency distributing questionnaires to existing internet panels designed to be representative of the population aged 18–75 years with regard to age and sex. This is useful information to repeat, as hospitalised patients may differ substantially from this characteristics profile and any difference in persistent symptoms or quality of life may partly reflect this. What is harder to quantify are the other less tangible differences between the separate cohorts providing controls and hospitalised/ICU patients.

5. The abstract concludes many former COVID-19 patients experience post-COVID symptoms at 24 months, but does not present those results in the abstract. This is the study's stated primary outcome (in the methods and the registered protocol), so this should be the focus of the numeric results throughout the manuscript, in addition to the secondary outcome measures noted.

6. The results section focuses on p-values. The prevalence of persistent symptoms in each group should be presented clearly with confidence intervals. Estimated differences between groups (either relative or absolute) should be presented alongside confidence intervals. This is all in keeping with best statistical practice and BMJ Open requirements.

7. Likewise, the mean EQ-5D scores (VAS and utility scores) should be presented with 95% confidence intervals. And as with prevalence, any comparisons should be presented as estimated difference and 95% confidence interval.

8. The study aims to describe the prevalence … of long COVID symptoms up to 2 years after acute infection and their relationship with health-related quality of life. What appears to be missing is quantification of quality of life in those who have long COVID compared to those who do not. Virtually everyone has been infected at least once, so comparison of those who have been infected with those who have not is unhelpful. Those who had not, probably have now, even if they didn't realise. What is probably more helpful, and more consistent with the primary aim, is quality of life amongst those who have had persistent symptoms for 2 years

vs those who recovered more quickly. i.e. what's are the utility scores for people with persistent long covid?

9. What's the justification for saying participants who report symptoms, but with symptom severity scores of less than 3 being labelled as not having the symptom? Why not a threshold of 2 or 4? Whilst validated measures won't have existed at the cohort inception, there has been plenty of time since to explore the psychometric properties and appropriate thresholds of illness.

10. Given that the controls and hospitalised/ICU cases come from different cohorts, I think that the statistical modelling is a little basic, just adjusting for age and sex. Given that the controls had an upper age limit of 75 that was apparently not applied to the cases, any adjustment for age maybe assumes linear associations extrapolate beyond the data collected in that group. Furthermore, differences in variant, vaccination status, comorbidities, may be relevant. I would like to see the directed acyclic graph used to justify which covariates to adjust for as confounders.

11. It's also usual in studies pooling individual participant data from cohorts to take the cohort into account in the models, often using a random/mixed-effects/multilevel approach. In your case, this might be problematic because controls all come from one cohort, which just goes to highlight the difficulty with combining cohorts with different sampling frameworks.

12. Backwards elimination is an inappropriate method for covariate selection. A quick google will suggest dozens of publications explaining why. I suggest this is corrected in any resubmission.

Minor comments

13. The abstract contains a large number of non-standard abbreviations that make it hard to follow.

14. "Patients" is an incorrect term for those participants who were not under the care of a doctor, e.g. non-hospitalised participants cannot be called patients here.

15. The abstract concludes that many former COVID-19 patients experience post-COVID symptoms at 24 months, but does not present those results in the abstract. This is the study's stated primary outcome (in registered protocol and the manuscript's methods), so the abstract should include these numeric results, with confidence intervals.

16. Rather than citing four publications in the introduction seemingly picked at random to justify the statement that around 50% of hospitalised patients experience persistent symptoms, the authors might look to existing meta-analyses, e.g. https://doi.org/10.1016/j.eclinm.2022.101762 (58% of hospitalised at >= 12 weeks) and/or https://doi.org/10.1093/ofid/ofad233 (45% of hospitalised at 12 weeks) experiencing at least one persistent symptom.

17. I'm not sure that I would label post-exertional malaise (more helpfully referred to as post-exertional symptom exacerbation) as "physical impairment" as it impacts more than just physical symptoms, e.g. cognitive dysfunction. Reported dizziness might indicate postural orthostatic tachycardia syndrome, which could be included alongside the other cardiovascular symptoms such as palpitations.

18. Pain has not been mentioned in the introduction other than abdominal pain and pain when breathing, yet muscle/joint pain is one of the more common persistent symptoms.

19. A little more detail on the EQ-5D-5L utility scores would be helpful in the methods, e.g. whether these use Dutch value sets, or borrows from other countries, or whether using a crosswalk from EQ-5D-3L. In addition, some comment in the results or discussion comparing to normative values, as these will be lower than 1.0, and/or other chronic conditions.

20. The manuscript should say stratified "by" not stratified "in" throughout.

21. "Is it possible that…" in strengths and limitations bullet points should be "It is possible that…"

**Reviewer**	**2**
**Name**	**Scholkmann, Felix**
**Affiliation**	**Universität Zürich**
**Date**	**09-Dec-2024**
**COI**	**None**

In their manuscript, Klein et al. present results from a longitudinal multiple cohort CORona Follow-Up (CORFU) study combining seven COVID-19 patient cohorts and a survey of the general population. The aim of the study was to investigate post-COVID symptoms 24 months after infection. As the design and analysis of the data have significant limitations and prevent a safe interpretation of the data, I cannot recommend publication of this manuscript.

Comments

1) Cases of SARS-CoV-2 infection were defined as positive PCR or CT scan and as "suspected cases". This is clearly inadequate as it allows for patients present in the dataset to have a "false positive" diagnosis for COVID-19, preventing reliable conclusions to be drawn about the prevalence of post-COVID-19 disease after confirmed (PCR) COVID-19. Only PCR positive COVID-19 must be included, all other cases (only CT positive or "suspected cases") must be excluded.

2) A large proportion of the subjects studied were vaccinated with the novel mRNA COVID-19 vaccines. As COVID-19 vaccination is associated with a variety of physiological effects

(acute and chronic) (which clearly overlap with the “post-COVID-19 syndrome”), it is a shortcoming of the study design not to control for this aspect. When comparing groups, the vaccination status must be exactly the same - to control that the "post-COVID symptoms" are not actually symptoms caused and/or modulated by the COVID-19 vaccine.

3) The authors must distinguish between "post-COVID symptoms" and "post-COVID-19 vaccination" symptoms within the 24-month time frame. This is possible as the vaccination status of the subjects is known. However, it appears that the authors did not ask the participants whether the symptoms were caused or modified by the COVID-19 vaccination and/or COVID-19 infection. Failure to ask this important question is another major flaw in the study design.

4) The comparison between the control group without COVID-19 and the other three groups is flawed because it is necessary to ensure that the control population is matched to the other three groups in terms of age and co-morbidities. To achieve this, three control groups need to be created for each of the three groups (non-hospitalised, hospitalised ward, hospitalised ICU) to test whether or not the questionnaires reflect post-COVID-19 symptoms.

5) The authors failed to differentiate between post-intensive care syndrome (PICS) and post-COVID-19 syndrome.

**Reviewer**	**3**
**Name**	**Marinho, Carolina**
**Affiliation**	**Universidade Federal de Minas Gerais**
**Date**	**24-Apr-2025**
**COI**	**None**

I commend the authors for presenting this important study on the long term follow up of post-COVID19 conditions. In the present manuscript, the authors describe the prevalence and severity of symptoms 2 years after initial infection, in a multiple cohort study. They also investigate HRQoL and its correlation with the persistent symptoms. The study protocol has been previously presented and the particularities of the current analysis is thoroughly reported.

The results were discussed extensively and satisfactorily. However, it would be interesting to delve deeper into the low frequency of cognitive problems found in the sample studied (page 14, lines 36-46). It seems to differ from those found in other studies with the same follow-up time. I would invite the authors to consider whether the following references could enrich their conclusions regarding their findings. Was it only due to the severity of the acute disease? Could there have been a progressive improvement in these symptoms in the population studied? If so, what factors would have contributed to this improvement?

JAMA Netw Open. 2022 Nov 1;5(11):e2242106. doi: 10.1001/jamanetworkopen.2022.42106. Post-COVID-19 Symptoms 2 Years After SARS-CoV-2 Infection Among Hospitalized vs Nonhospitalized Patients. César Fernández-de-Las-Peñas et al.

Journal of Infection, 2024 Volume 88, Issue 2, 77 – 88. Persistence of post-COVID symptoms in the general population two years after SARS-CoV-2 infection: A systematic review and meta-analysis. Fernandez-de-las-Peñas, Cesar et al.

Rev Neurol. 2025 Jan 24;79(12):37385. doi: 10.31083/RN37385. Cognition and Long COVID: A PRISMA Systematic Review of Longitudinal Studies. María Alejandra Tudorache Pantaz

Small fixes:

On page 13, lines 46-47, the first sentence of the paragraph seems redundant or truncated.

On page 15, lines 29 to 40, the authors could make it clearer which work they are referring to – the Chinese paper or their own. Furthermore, in lines 33 and 34, it is not clear which EQ-VAS and utility score they are referring to.

The sum of participants at 24months in Table 1 is 901 (146 + 509 + 246), different from 904 in results (page 12, line 45).

There is a blank column in Table 2.

**Reviewer**	**4**
**Name**	**Leite, Arnaldo**
**Affiliation**	**Federal University of Minas Gerais, Departamento de Clínica Médica**
**Date**	**25-Apr-2025**
**COI**	**None**

Dear authors,

Congratulations to everyone involved on the study and the effort in working on such a prevalent topic. The post-covid condition still does not have a well-defined conceptualization, and its complexity makes the study even more arduous. This is a very relevant topic for the current moment. To contribute to the article, I have some comments and suggestions that I would like the authors to evaluate.

2. Is the abstract accurate, balanced and complete?

The abstract is adequate; however, I suggest attention to the following sentence in the abstract: “Participants: Former COVID-19 patients and healthy controls participated in this cohort study.” The term “healthy controls” is inadequate because they were controls who

supposedly did not present COVID-19. Controls without COVID-19 are actually controls without a previous history of COVID-19, but they may have other diseases, as specified in the body of the article, such as the prevalence of asthma among the patients in the control group.

3. Is the study design appropriate to answer the research question?

Yes, but some methodological aspects need to be better clarified, as per the attached document.

4. Are the methods described sufficiently to allow the study to be repeated?

In the study, post-COVID symptoms were analyzed up to 24 months after infection. The assessment was done at several times. There may have been some selection bias, which needs to be thoroughly explored in the discussion of the study's limitations. Other methodological aspects should be better clarified, as per the attached file.

10. Are the results presented clearly?

No. I suggest clarifying some items as per the attached file.

11. Are the discussion and conclusions justified by the results?

No. I suggest clarifying some items as per the attached file.

12. Are the study limitations discussed adequately?

In limitations, it is stated that "Is it possible that the control group contained more cases of (asymptomatic) COVID-19 patients than identified, as people may have contracted COVID-19 without noticing. This could have resulted in some misclassification of controls." It is written inadequately because the control group was composed of patients who supposedly did not have COVID-19. In fact, the control group, as it was composed, could have some unidentified case of COVID-19.

Other aspects related to limitations should be better clarified and better discussed as limitations, as per the attached file.

thanks for considering.

The best!

The comments below follow the sequence presented in the manuscript.

Regarding the article's strengths, it is mentioned that:

1) "The long follow-up period up until 24 months after infection with the SARS-CoV-2 virus allows for estimating long-term post-COVID symptom prevalence and severity." Evaluate whether this is indeed a strong aspect of the study, since the percentage of responders at 24 months was low.

2) “By grouping several cohorts, we were able to include a diverse population of patients and controls that enabled us to provide information on diverse groups based on sex, BMI, age, and severity of disease” – this sentence is incorrect because the patients in the control group were participants in only one cohort. Furthermore, the severity of the acute condition was only attested by ICU admission, without considering other relevant aspects that categorize the patient's severity.

3) “The control population helps to shed light on the prevalence and severity of the same symptoms that are used to diagnose post-COVID condition” Verify if this information is true. Were associated conditions, such as depression, past viral infection, or comorbidities, investigated in the control group patients? Could the participation of individuals in the cohort be biased?

In the introduction, it is mentioned that:

1) “Post-COVID condition refers to a range of symptoms that persist for at least 3 months after the acute phase of the COVID-19 disease” – modify the text to symptoms that persist or that begin...

2) “Our study aim was to assess the (excess) prevalence and severity of post-COVID symptoms from 3 months up to 2 years after SARS-CoV-2 infection and compare symptoms to controls without known SARS-CoV-2 infection in the past” - There is a great variability of time, that is, from 3 months to 2 years. The manuscript title refers to 2 years later – wouldn't it be more appropriate to write “over 2 years”? Was the severity of the symptoms actually evaluated? Wouldn't it be better to evaluate the effect of the symptoms on quality of life?

In methodology:

1) “Data from the following cohorts were combined: the Maastricht Intensive Care COVID (MaastrICCht) cohort [17, 18], the Bernhoven early detection of vascular damage after COVID-19 cohort (COVAS) cohort [19], the ZuydErLand COVID-19 regiStry (ELVIS) cohort and the Cardiac complications in patients with COVID-19 (CAPACITY-COVID) cohort [21] and the community-based POPulation health impact of the COVID-19 pandemic (POPCORN cohort [22].” – It is reported 7 cohorts, but only 4 cohorts appear here in the methodology.

2) “POPCORN participants who reported to have suffered from (mild) COVID-19 were counted as cases” – this is an important limitation of the study that needs to be discussed, as positivity could not be proven.

3) “Participants in all cohorts had to be at least 18 years of age, and SARS-CoV-2 cases were either confirmed by PCR or CT-scan (COVID-19 Reporting and Data System (CO-RADS) with a score of 4-5) or were suspected cases, as there was limited testing capacity at the start of the pandemic.” Here, a possible bias is observed, as some cases were not confirmed. It would be relevant to inform the percentage of those considered suspect cases, but without laboratory confirmation. Should only suspect cases be excluded?

4) “Former COVID-19 patients and non-COVID-19 controls were sent one or more questionnaires (depending on the cohort) on symptoms associated with post-COVID For peer review only condition” – but was there no standardization? One or more questionnaires?

5) “CORFU participants were divided into four subgroups: 1) participants without a known SARS-CoV-2 infection, i.e., the controls, 2) participants with proven SARS-CoV-2 infection without hospitalization” – rewrite this differently, as suspect cases cannot be considered confirmed cases.

6) “...4) participants with SARS-CoV-2 infection with intensive care unit (ICU)” – was there no analysis of a subgroup with mechanical ventilation, or with hemodynamic dysfunction, or with any other severity criterion?

7) “SARS-CoV-2 infection with intensive care unit (ICU) admission. Participants of the cohorts were invited to complete the CORFU questionnaire at 3, 6, 12, 18, and 24 months after initial SARS-CoV-2 infection” – it is necessary to specify how the invitation to respond to the questionnaire was made. Contact via email? In person during an outpatient visit?

8) “The timing of the CORFU questionnaire was determined by the participant’s date of first infection (diagnosis and/or admission). Due to the timing of study initiation, some participants could not complete earlier questionnaires, depending on the cohort; most participants completed either 1 or 2 during follow-up.” This is already a result, not methodology. An important possible bias, as most completed only 1 or 2 questionnaires.

9) “Depending on the cohort and patient preference, questionnaires were completed either digitally or on paper” – again, how were the patients contacted? By email? Another way?

Data Collection:

1) "Headache as a symptom was added to the CORFU questionnaire after June 28, 2022, as early studies reported headache as a symptom associated with post-COVID condition." Was the percentage of questionnaires that included this variable specified?

2) "In addition, we asked for vaccination status (having had one or more versus none)." What is the relevance of this? Was it indicated when the patient received the vaccine?

3) "Second, we dichotomised symptoms associated with post-COVID condition into being present or absent. A symptom was registered as 'present' when it was scored at least moderately severe (3 or above on a 5-point Likert scale)." Better specify whether symptoms were considered existent only with a score of 3 or higher. What is the reason for this criterion? Why disregard patients with milder symptoms?

4) "We computed the percentage of patients vaccinated at the time of completing their 24-month questionnaire and computed the percentage of vaccinated and unvaccinated patients that exhibited post-COVID symptoms, stratified by the four subgroups." Check if vaccination status is included in the study objectives. It doesn't make much sense, as it does not analyze when the vaccine was administered.

In Results:

1) "Incluímos 4.291 participantes que completaram um total de 5.523 questionários (Tabela 1). Isso incluiu 3.086 (72,0%) controles não COVID." We notice a high percentage of controls with few questionnaires answered by the number of participants. This can generate bias, as only patients who persisted with symptoms may have continued to answer the questionnaires.

2) "We received the most questionnaires from former COVID-19 patients on the 24-month follow-up moment, i.e., 904." It may be that there was a tendency to answer the questionnaire if the symptom was persistent.

3) "The mean age was lower in the control group…" - Possible selection bias, as the controls are younger.

4) The male-to-female ratio was close to equality in the control group (48.7% male), but in the clinical subgroups, there were substantially more males than females, with the highest percentage of males in the ICU subgroup. This difference in gender can impact, as it is known that post-COVID symptoms are more prevalent in women.

5) In the control group and the subgroup of non-hospitalized patients, asthma was the most frequently reported chronic disease (11.5% and 10.9%, respectively). For ward patients, it was diabetes and arrhythmia or palpitations, followed closely by osteoarthritis and low back pain. In addition to being younger, the control group was also characterized by milder comorbidities. Another potential bias. Was this corrected? There was no matching of cases for the control group patients.

6) "For the ward patients, this was diabetes and arrhythmia or palpitations, followed closely by osteoarthritis and low back pain" - strange palpitations and low back pain placed as comorbidities. Also, arrhythmia disease as a comorbidity may reveal inclusion bias of patients in a cohort that evaluated cardiac patients.

7) It seems that the symptoms were judged for severity by the Likert scale. There is a need to clarify whether the presence of symptoms was defined by the scale (3 or more).

8) "Of all former COVID-19 patients, 89.0% had been vaccinated at least once at 24 months. We observed large differences in vaccination between rates across subgroups. The lowest rates were found in the non-hospitalised patient group (67.1%) and the highest in the patients admitted to the ward (95.7%). In the former ICU patients, this was 87.9% (p<0.001). At 24 months, 43.9% of vaccinated non-hospitalised patients had at least one post-COVID symptom present compared to 39.6% of non-vaccinated non-hospitalised patients (p= 0.753), 65.3% of vaccinated patients admitted to the ward had at least one symptom compared to 59.1% for non-vaccinated patients (p=0.712), and 64.1% of ICU patients had at least one symptom present compared to 56.7% for non-vaccinated ICU patients (p=0.559)" Among all former COVID-19 patients, 89.0% had been vaccinated at least once at 24 months. We observed large differences in vaccination between the rates across subgroups. The lowest rates were found in the non-hospitalised patient group (67.1%) and the highest in the patients admitted to the ward (95.7%). In the former ICU patients, this was 87.9% (p<0.001). At 24 months, 43.9% of vaccinated non-hospitalised patients had at least one post-COVID symptom present compared to 39.6% of non-vaccinated non-hospitalised patients (p=0.753), 65.3% of vaccinated patients admitted to the ward had at least one symptom compared to 59.1% for non-vaccinated patients (p=0.712), and 64.1% of ICU patients had at least one symptom present compared to 56.7%

for non-vaccinated ICU patients (p=0.559)". This vaccination analysis is irrelevant as the vaccination timing was not checked whether before the acute infection or after, and at what point. It is not possible to verify any association between vaccination and post-COVID symptoms or the severity of the acute illness, whether hospitalization was needed or not. Were the controls analyzed for vaccination?

9) "At 24 months after infection, the mean and standard deviation (SD) of self-rated health on the EQ-VAS were 71.6 (17.9) for non-hospitalized patients and 70.0 (17.3) and 71.4 (17.5) for participants admitted to the ward or ICU, respectively. This was significantly lower than the control group mean EQ-VAS at 75.6 (17.7) (p<0.001 for controls versus former COVID-19 patients)…" - It was lacking to inform if there was a difference among the COVID groups.

In Discussion:

1) "Possible cognitive dysfunction was not related to the SARS-CoV-2 infection but post-intensive care syndrome (PICS) could have played a role in this. Also, since the ICU's were full, these patients had to stay at the general ward. Thus the distribution on disease severity we made could have led to a distorted picture." – Review this last sentence, as such distortion would not impact the increase in the prevalence of cognitive changes in ICU patients.

2) "Symptoms 24 months after acute infection between the former ward and ICU patients differed only slightly, although treatment in the ICU differed (patients with SARS-Cov-2 were sedated and mechanically ventilated during their ICU admission)." Is this difference not shown? Correct the text, as a patient in the ICU is not necessarily subjected to mechanical ventilation.

3) "Although we expected that the EQ-5D-5L and VAS results would be lower at 24 months due to the severity during the acute phase for former ICU patients compared to patients who had not been admitted to the hospital, this was not the case in our study. The results indicate that the severity of the acute SARS-CoV-2 infection alone doesn't not predict HRQOL at the long term." The text is confusing, as the discussion indicates that several severe patients stayed in the ward because the ICU was full. The severity cannot be measured solely by ICU admission (was any other severity score used? SOFA, SAPS, APACHE, use of mechanical ventilation, need for vasoactive drugs?).

4) "These risk factors may contribute to further improving the definition and prediction of post-COVID condition" Correct this sentence since I understand that risk factors cannot contribute to improving the definition of a condition.

5) "Regarding vaccination status, patients admitted to the hospital had the highest and non-hospitalised patients with the lowest vaccination rate. Since most of the patients in our study population had a SARS-CoV-2 infection before the vaccination rounds started, we could not determine, that any causal relation between vaccination status and long term symptoms." I understand that the vaccination variable should not be analyzed for this reason.

In Strengths and Limitations:

1) "This study has several strengths. First, the long follow-up period up until 24 months after infection with the SARS-CoV-2 virus allows for estimating long-term post-COVID symptom prevalence and severity." This statement is not entirely true, as the percentage of patients "followed up" for a long time was small. As commented earlier, some bias may have occurred. What was the follow-up like? Were they invited to answer the questionnaire by phone?

2) "Also, by grouping several cohorts, we were able to include a diverse population of patients and controls that enabled us to provide information on diverse groups based on sex, BMI, age, and severity of disease. The control population helps to shed light on the prevalence and severity of the same symptoms that are used to diagnose post-COVID condition. However, the control group was slightly younger on average, more often highly educated, and had fewer chronic diseases." There was no matching, this greatly hurts this conclusion. Therefore, this is not a strength; it is more of a limitation.

3) "..This could have resulted in underestimating the number of patients with severe complaints" add: led the patient not to respond to the questionnaire.

4) "...we did not have many participants with multiple questionnaires during follow-up. This would have allowed for the estimation of within-person change." Add that the percentage of the universe that responded at 24 months was very small.

thanks for considering.

The best!

VERSION 1 - AUTHOR RESPONSE

Reviewer: 1
Dr. Darren Greenwood, University of Leeds
Comments to the Author:

General comments
1. This is a hugely important topic. A large and growing number of people have now been living with long covid for over two years. Reported quality of life is measurable worse than many better understood chronic conditions, but with relatively little research into what to do about it. The impact on those individuals' lives is substantial. The impact on reduced workforce and increased benefit payments is also significant.
2. This paper offers the potential to track persistence of symptoms in the same cohort over 2 years, though follow-up is poor and repeated measures few.

Major comments
3. The study benefits from pooling rich individual participant data from seven longitudinal cohort studies, but the total sample size remains quite small for a prevalence study across subgroups, presumably because of the longitudinal nature of the data collection and subsequent losses to follow-up. Initial response rates have not been presented, only the numbers included at each time point. Losses to follow-up are

quantified and shown to be around 90%, leaving substantial room for bias. There is strong potential for people with persistent symptoms to be more motivated to respond, or less able to respond, and for this to be different between the groups.
For this study, questionnaires were sent in a predefined period of calendar time. Therefore, most participants only completed one or two questionnaires. We did not follow all participants from onset of COVID-19 to two years after. Therefore, most respondents at different timepoints are unique; others are not lost to follow up, but in another phase after their initial disease.

4. The study benefits from a healthy control sample missing from many similar studies. The participants appears to be dominated by the two larger cohorts (CAPACITY-COVID and POPCOrn), with controls appearing to come just from POPCOrn, but hospitalised and ICU patients from the other cohorts. The sampling methods of the controls is unclear in both the manuscript and the protocol paper referenced. The reference within the protocol points to a manuscript describing recruitment by an international market research agency distributing questionnaires to existing internet panels designed to be representative of the population aged 18–75 years with regard to age and sex. This is useful information to repeat, as hospitalised patients may differ substantially from this characteristics profile and any difference in persistent symptoms or quality of life may partly reflect this. What is harder to quantify are the other less tangible differences between the separate cohorts providing controls and hospitalised/ICU patients.
The recruitment strategies of clinical cohorts were similar for all but the MaastrICCht cohort. Those cohorts recruited participants who came to the hospital with COVID-19 complaints and were subsequently tested using the diagnostic criteria at that time. Based on severity, they were either admitted to the hospital or sent home. Patients who were admitted would either be admitted to the ward or ICU, or to the ward and later the ICU, depending on progression and availability of ICU beds. No further exclusion criteria were applied, making these cohorts comparable. Combined, there is a mix of types of hospitals (e.g. non-academic, academic, tertiary referral), aiding generalizability of our results. The MaastrICCht cohort included only participants that had been admitted to the ICU. For this reason, we additionally stratified analyses based on admission status (not admitted, admitted (only) to the ward, admitted (also) to the ICU).

5. The abstract concludes many former COVID-19 patients experience post-COVID symptoms at 24 months but does not present those results in the abstract. This is the study's stated primary outcome (in the methods and the registered protocol), so this should be the focus of the numeric results throughout the manuscript, in addition to the secondary outcome measures noted.
We have added this information to the abstract.

6. The results section focuses on p-values. The prevalence of persistent symptoms in

each group should be presented clearly with confidence intervals. Estimated differences between groups (either relative or absolute) should be presented alongside confidence intervals. This is all in keeping with best statistical practice and BMJ Open requirements.
We have added a statement on computing 95% confidence intervals in the methods section. Furthermore, we have added 95% confidence intervals to the results section that discusses inferential results.

7. Likewise, the mean EQ-5D scores (VAS and utility scores) should be presented with 95% confidence intervals. And as with prevalence, any comparisons should be presented as estimated difference and 95% confidence interval.
We have added this information to the results.

8. The study aims to describe the prevalence … of long COVID symptoms up to 2 years after acute infection and their relationship with health-related quality of life. What appears to be missing is quantification of quality of life in those who have long COVID compared to those who do not. Virtually everyone has been infected at least once, so comparison of those who have been infected with those who have not is unhelpful. Those who had not, probably have now, even if they didn't realise. What is probably more helpful, and more consistent with the primary aim, is quality of life amongst those who have had persistent symptoms for 2 years vs those who recovered more quickly. i.e. what's are the utility scores for people with persistent long covid?
In the revised version of our manuscript, we have added a quantification of quality-of-life measures (both EQ-VAS and utility) for those with long COVID and those without, including 95% confidence intervals, and computed the mean differences between groups.
Due to the limited period of calendar time that questionnaires were sent, we do not have longitudinal trajectories of participants of more than two timepoints. As the time of initial infection differs between participants, some participants may have completed the 6- and 12-month questionnaires, whereas others may have other combinations of questionnaires. For that reason, we were not able to assess persistent long COVID.

9. What's the justification for saying participants who report symptoms, but with symptom severity scores of less than 3 being labelled as not having the symptom? Why not a threshold of 2 or 4? Whilst validated measures won't have existed at the cohort inception, there has been plenty of time since to explore the psychometric properties and appropriate thresholds of illness.
The cut-off value was decided in a consensus meeting with members of the EuroQol foundation, who developed the EQ-5D-5L and also the additional symptom questionnaires. We agree that to some extent, any choice for a cut-off value would be arbitrary. The one we used may have misclassified participants with symptoms as

having none, because their symptoms were relatively mild. We preferred this over classifying anyone with even very mild symptoms as positive.
At present, no studies on the psychometrics of these specific so-called ´bolt on' questions for post COVID have been performed. We recently obtained a grant to study these. However, that is an ongoing project for the next two years, and we feel that the results of the current study are too important to wait for such evidence to be published. In another paper submitted at present, we looked at clustering patients according to their symptoms, and at predicting post-COVID for individual participants. For these analyses, it made sense to change the cut-off values to assess the robustness of the conclusions. We concluded that the cut-off value may have a substantial effect on prevalence, but other analyses yield similar conclusions regardless of whether 2 or 3 was used as cut-off (note that we did not use 4 as the prevalence of such severe symptoms was relatively low).

10. Given that the controls and hospitalised/ICU cases come from different cohorts, I think that the statistical modelling is a little basic, just adjusting for age and sex. Given that the controls had an upper age limit of 75 that was apparently not applied to the cases, any adjustment for age maybe assumes linear associations extrapolate beyond the data collected in that group. Furthermore, differences in variant, vaccination status, comorbidities, may be relevant. I would like to see the directed acyclic graph used to justify which covariates to adjust for as confounders.
We have made a directed acyclic graph to assess whether we were currently adjusting for the correct set of confounders (see figure below). Upon review, we have added the list of comorbidities to the adjustment set. Note that BMI is not part of the graph as BMI was not recorded in each of our pre-defined groups. Both the methods and results (i.e. Table 3) have been updated accordingly.

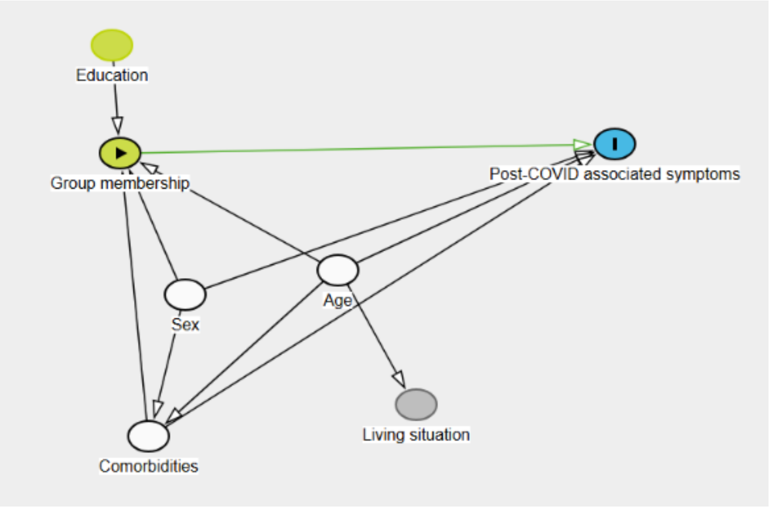

11. It's also usual in studies pooling individual participant data from cohorts to take the cohort into account in the models, often using a random/mixed-effects/multilevel

approach. In your case, this might be problematic because controls all come from one cohort, which just goes to highlight the difficulty with combining cohorts with different sampling frameworks.
We had refrained from using multilevel modeling not just because of controls coming from the same cohort, but also because the hospital cohorts had very similar recruitment strategies and used the same source population. In essence, the combined cohorts may be seen as a multicenter study.

12. Backwards elimination is an inappropriate method for covariate selection. A quick google will suggest dozens of publications explaining why. I suggest this is corrected in any resubmission.
We agree with the reviewer on the use of backward elimination. The results as submitted before were results of fully adjusted models, not only with covariates kept after elimination. The methods section erroneously stated the use of that technique and has now been omitted from the text.

Minor comments

13. The abstract contains a large number of non-standard abbreviations that make it hard to follow.
We agree with the reviewer that the abstract might be confusing due to the large number of non-standard abbreviations and we have adapted this accordingly.

14. “Patients” is an incorrect term for those participants who were not under the care of a doctor, e.g. non-hospitalised participants cannot be called patients here.
We have used the term patient to refer to all individuals who had been infected with the SARS-CoV-2 virus, whether hospitalised or not. To distinguish between these individuals and those in the control population, the latter are referred to as participants.

15. The abstract concludes that many former COVID-19 patients experience post-COVID symptoms at 24 months but does not present those results in the abstract. This is the study’s stated primary outcome (in registered protocol and the manuscript’s methods), so the abstract should include these numeric results, with confidence intervals.
We agree with the reviewer, we have added this information to the abstract.

16. Rather than citing four publications in the introduction seemingly picked at random to justify the statement that around 50% of hospitalised patients experience persistent symptoms, the authors might look to existing meta-analyses, e.g. https://doi.org/10.1016/j.eclinm.2022.101762 (58% of hospitalised at >= 12 weeks) and/or https://doi.org/10.1093/ofid/ofad233 (45% of hospitalised at 12 weeks) experiencing at least one persistent symptom.
Thank you for this useful literature, we have added these to the introduction.

17. I'm not sure that I would label post-exertional malaise (more helpfully referred to as post-exertional symptom exacerbation) as "physical impairment" as it impacts more than just physical symptoms, e.g. cognitive dysfunction. Reported dizziness might indicate postural orthostatic tachycardia syndrome, which could be included alongside the other cardiovascular symptoms such as palpitations.
Thanks for this insighful comment, this has been adapted in the manuscript.

18. Pain has not been mentioned in the introduction other than abdominal pain and pain when breathing, yet muscle/joint pain is one of the more common persistent symptoms.
Thanks for this insighful comment, this has been adapted in the manuscript.

19. A little more detail on the EQ-5D-5L utility scores would be helpful in the methods, e.g. whether these use Dutch value sets, or borrows from other countries, or whether using a crosswalk from EQ-5D-3L. In addition, some comment in the results or discussion comparing to normative values, as these will be lower than 1.0, and/or other chronic conditions.
We have added more details on the EQ-5D-5L in the methods section, and also we have commented in the discussion on the comparison to normative values.

20. The manuscript should say stratified "by" not stratified "in" throughout.
This has been adapted in the manuscript.

21. "Is it possible that…" in strengths and limitations bullet points should be "It is possible that…"
This has been changed in the manuscript.

Reviewer: 2
Dr. Felix Scholkmann, Universität Zürich
Comments to the Author:
In their manuscript, Klein et al. present results from a longitudinal multiple cohort CORona Follow-Up (CORFU) study combining seven COVID-19 patient cohorts and a survey of the general population. The aim of the study was to investigate post-COVID symptoms 24 months after infection. As the design and analysis of the data have significant limitations and prevent a safe interpretation of the data, I cannot recommend publication of this manuscript.

Comments
1) Cases of SARS-CoV-2 infection were defined as positive PCR or CT scan and as "suspected cases". This is clearly inadequate as it allows for patients present in the

dataset to have a "false positive" diagnosis for COVID-19, preventing reliable conclusions to be drawn about the prevalence of post-COVID-19 disease after confirmed (PCR) COVID-19. Only PCR positive COVID-19 must be included, all other cases (only CT positive or "suspected cases") must be excluded.
Most participants in our cohorts were recruited right at the start of the pandemic. At that time, diagnostic criteria were only just being developed and changed throughout the subsequent waves of the pandemic. In some cases, PCR testing was only very limited available and therefore not applied to patients in whom the diagnosis COVID-19 was considered very likely, not only based on e.g. CT criteria, but also on anamnesis. PCR-negative patients were regarded as not having COVID-19. We think that classifying those that were not tested using a PCR test but were regarded as positive usng other diagnostic criteria considered accurate at the time, as negative for this study, will result in severe misclassification.

2) A large proportion of the subjects studied were vaccinated with the novel mRNA COVID-19 vaccines. As COVID-19 vaccination is associated with a variety of physiological effects (acute and chronic) (which clearly overlap with the “post-COVID-19 syndrome”), it is a shortcoming of the study design not to control for this aspect. When comparing groups, the vaccination status must be exactly the same - to control that the "post-COVID symptoms" are not actually symptoms caused and/or modulated by the COVID-19 vaccine.
Thank you for pointing this out. The question on vaccination status was added only at a later stage in calendar time. We do not know for many participants when their (multiple) vaccinations were received relative to them completing our follow-up questionnaires. For that reason, it is not possible for us to stratify based on vaccination status or to adjust in the models that we use to compare groups. We have added this to the limitations section of the discussion.

3) The authors must distinguish between "post-COVID symptoms" and "post-COVID-19 vaccination" symptoms within the 24-month time frame. This is possible as the vaccination status of the subjects is known. However, it appears that the authors did not ask the participants whether the symptoms were caused or modified by the COVID-19 vaccination and/or COVID-19 infection. Failure to ask this important question is another major flaw in the study design.
Thank you for pointing this out. We have added a part in our discussion section that elaborates on this limitation.

4) The comparison between the control group without COVID-19 and the other three groups is flawed because it is necessary to ensure that the control population is matched to the other three groups in terms of age and co-morbidities. To achieve this, three control groups need to be created for each of the three groups (non-hospitalised,

hospitalised ward, hospitalised ICU) to test whether or not the questionnaires reflect post-COVID-19 symptoms.
From an epidemiolocal point of view, matching is inferior to multivariable adjustment. Matching can increase the risk of bias considerably and may have a substantial effect on precision of statistical estimates and statistical power as not all participants will be able to be matched. For these reasons, we adjusted between-group differences for potential confounding factors. Please see e.g. doi: 10.1007/s10654-023-01046-9.

5) The authors failed to differentiate between post-intensive care syndrome (PICS) and post-COVID-19 syndrome.
We acknowledge that there is a substantial overlap in symptoms of both PICS and post-COVID. Many patients with a post-COVID diagnosis (not confined to our study, but in daily practice as well) who had been admitted to an ICU may be misclassified as such. Part of our CORFU study team are the intensivists who treated the patients who were subsequently enrolled in our study, and we shared many thoughts on this. In the end, we concluded it was not possible to discriminate these two from each other, and classifying patients as having post-COVID would most accurately reflect daily practice.

Reviewer: 3
Dr. Carolina Marinho, Universidade Federal de Minas Gerais
Comments to the Author:
1. I commend the authors for presenting this important study on the long term follow up of post-COVID19 conditions. In the present manuscript, the authors describe the prevalence and severity of symptoms 2 years after initial infection, in a multiple cohort study. They also investigate HRQoL and its correlation with the persistent symptoms. The study protocol has been previously presented and the particularities of the current analysis is thoroughly reported.
The results were discussed extensively and satisfactorily. However, it would be interesting to delve deeper into the low frequency of cognitive problems found in the sample studied (page 14, lines 36-46). It seems to differ from those found in other studies with the same follow-up time. I would invite the authors to consider whether the following references could enrich their conclusions regarding their findings. Was it only due to the severity of the acute disease? Could there have been a progressive improvement in these symptoms in the population studied? If so, what factors would have contributed to this improvement?
JAMA Netw Open. 2022 Nov 1;5(11):e2242106. doi: 10.1001/jamanetworkopen.2022.42106. Post-COVID-19 Symptoms 2 Years After SARS-CoV-2 Infection Among Hospitalized vs Nonhospitalized Patients. César Fernández-de-Las-Peñas et al.
Journal of Infection, 2024 Volume 88, Issue 2, 77 – 88. Persistence of post-COVID symptoms in the general population two years after SARS-CoV-2 infection: A systematic

review and meta-analysis. Fernandez-de-las-Peñas, Cesar et al.
Rev Neurol. 2025 Jan 24;79(12):37385. doi: 10.31083/RN37385. Cognition and Long COVID: A PRISMA Systematic Review of Longitudinal Studies. María Alejandra Tudorache Pantaz
We thank the reviewer for this interesting papers, we have read them with great interest. However, we currently lack the data required to gain a deeper understanding of the low prevalence of cognitive problems in our study population.

2. Small fixes:
On page 13, lines 46-47, the first sentence of the paragraph seems redundant or truncated.
On page 15, lines 29 to 40, the authors could make it clearer which work they are referring to – the Chinese paper or their own. Furthermore, in lines 33 and 34, it is not clear which EQ-VAS and utility score they are referring to.
The sum of participants at 24months in Table 1 is 901 (146 + 509 + 246), different from 904 in results (page 12, line 45).
There is a blank column in Table 2.
Thank you for pointing this out, we have adapted this in the text. Also, we adjusted Table 1 to reflect the actual number of participants at 24 months (i.e. 904 instead of 901).

Reviewer: 4 (see also attached)
Dr. Arnaldo Leite, Federal University of Minas Gerais
Comments to the Author:
Dear authors,
Congratulations to everyone involved on the study and the effort in working on such a prevalent topic. The post-covid condition still does not have a well-defined conceptualization, and its complexity makes the study even more arduous. This is a very relevant topic for the current moment. To contribute to the article, I have some comments and suggestions that I would like the authors to evaluate.

2. Is the abstract accurate, balanced and complete?
The abstract is adequate; however, I suggest attention to the following sentence in the abstract: “Participants: Former COVID-19 patients and healthy controls participated in this cohort study.” The term “healthy controls” is inadequate because they were controls who supposedly did not present COVID-19. Controls without COVID-19 are actually controls without a previous history of COVID-19, but they may have other diseases, as specified in the body of the article, such as the prevalence of asthma among the patients in the control group.
We agree with the reviewer and we have adapted this in the manuscript.

3. Is the study design appropriate to answer the research question?

Yes, but some methodological aspects need to be better clarified, as per the attached document.
These points are addressed separately.

4. Are the methods described sufficiently to allow the study to be repeated?
In the study, post-COVID symptoms were analysed up to 24 months after infection. The assessment was done at several times. There may have been some selection bias, which needs to be thoroughly explored in the discussion of the study's limitations. Other methodological aspects should be better clarified, as per the attached file.

5. Are the results presented clearly?
No. I suggest clarifying some items as per the attached file.
These points will be addressed separately.

6. Are the discussion and conclusions justified by the results?
No. I suggest clarifying some items as per the attached file.
These points will be addressed separately.

7. Are the study limitations discussed adequately?
In limitations, it is stated that "Is it possible that the control group contained more cases of (asymptomatic) COVID-19 patients than identified, as people may have contracted COVID-19 without noticing. This could have resulted in some misclassification of controls." It is written inadequately because the control group was composed of patients who supposedly did not have COVID-19. In fact, the control group, as it was composed, could have some unidentified case of COVID-19.
Thanks for this insight, we have changed this in the limitations.

Other aspects related to limitations should be better clarified and better discussed as limitations, as per the attached file.
thanks for considering.
The best!

<u>Comments in attachment</u>

Dear authors,

Congratulations to everyone involved on the study and the effort in working on such a prevalent topic. The post-covid condition still does not have a well-defined conceptualization, and its complexity makes the study even more arduous. This is a very relevant topic for the current moment. To contribute to the article, I have some comments and suggestions that I would like the authors to evaluate.

The comments below follow the sequence presented in the manuscript.

Regarding the article's strengths, it is mentioned that:

1. “The long follow-up period up until 24 months after infection with the SARS-CoV-2 virus allows for estimating long-term post-COVID symptom prevalence and severity.” Evaluate whether this is indeed a strong aspect of the study, since the percentage of responders at 24 months was low.

Because of the nature of data collection (i.e. a brief period of calendar time), patients were only sent questionnaires that matched their time-since-infection during that period. For that reason, it may seem that the response rate is low (e.g. only 904 at 24 months). However, many other participants never received a 24-month questionnaire as they did not reach this follow-up time before the data collection period ended.

2. “By grouping several cohorts, we were able to include a diverse population of patients and controls that enabled us to provide information on diverse groups based on sex, BMI, age, and severity of disease” – this sentence is incorrect because the patients in the control group were participants in only one cohort. Furthermore, the severity of the acute condition was only attested by ICU admission, without considering other relevant aspects that categorize the patient's severity.

We have changed the sentence to: “Also, by grouping several cohorts and data from a survey...” . Furthermore, we acknowledge that home, admission to the ward, and admission to the ICU is only a proxy for COVID-19 severity. However, there is no gold standard that exists to classify participants into severity groups. In some cases, patients may not have been admitted to the ICU as their prognosis was very severe and the choice was made for the ward so that at least family members could visit. However, these patients likely did not survive and hence, were not misclassified in this study. However, we do acknowledge other types of misclassification may have occured due to e.g. differences between hospitals. For that reason, we kept all group labels (e.g. non-hopsitalized, hospitalized (ICU) etc.) instead of renaming into e.g. “mild” and “severe”.

3. “The control population helps to shed light on the prevalence and severity of the same symptoms that are used to diagnose post-COVID condition” Verify if this information is true. Were associated conditions, such as depression, past viral infection, or comorbidities, investigated in the control group patients? Could the participation of individuals in the cohort be biased?

Control participants were asked to complete questionnaires that were in large part the same as the questionnaires members of the patient cohorts received (with the exception of course of the disease-specific questions). Hence, these participants also scored the same 5-point Likert scales on symptoms that are associated with post-COVID but are not specific to the syndrome. All between-group null-hypothesis tests were adjusted for, amongst other covariates, all comorbidities. Therefore, we view the

control cohort as a "baseline" of these symptoms in a randomly selected sample of whom those who did experience COVID-19 were excluded (i.e. transferred to one of the other groups in our study).

In the introduction, it is mentioned that:

1. "Post-COVID condition refers to a range of symptoms that persist for at least 3 months after the acute phase of the COVID-19 disease" – modify the text to symptoms that persist or that begin...

We have adapted this sentence in the introduction.

2. "Our study aim was to assess the (excess) prevalence and severity of post-COVID symptoms from 3 months up to 2 years after SARS-CoV-2 infection and compare symptoms to controls without known SARS-CoV-2 infection in the past" - There is a great variability of time, that is, from 3 months to 2 years. The manuscript title refers to 2 years later – wouldn't it be more appropriate to write "over 2 years"? Was the severity of the symptoms actually evaluated? Wouldn't it be better to evaluate the effect of the symptoms on quality of life?

We agree with the reviewer that the title of our manuscript may cause some unclarity, we have adapted this to address this point.

<u>In methodology:</u>

1. "Data from the following cohorts were combined: the Maastricht Intensive Care COVID (MaastrICCht) cohort [17, 18], the Bernhoven early detection of vascular damage after COVID- 19 cohort (COVAS) cohort [19], the ZuydErLand COVID-19 regiStry (ELVIS) cohort and the Cardiac complications in patients with COVID-19 (CAPACITY-COVID) cohort [21] and the community- based POPulation health impact of the COVID-19 pandemic (POPCORN cohort [22]." – It is reported 7 cohorts, but only 4 cohorts appear here in the methodology.

That is correct, as is shown by figure 1 there are 7 cohorts in this study. . However, for this paper we only have used data from 5(!) cohorts. For one, the Adelante cohort will only be used for manuscripts on rehabilitation *of patients with post-COVID, as they were sources in a different way than the other clinical cohorts. The other missing cohort had much overlap in the patient sample from CAPACITY, and collected additional clinical data of those patients, irrelevant to this manuscript. In figure 1 we have added a * to flag which cohorts were used for the analysis in this paper*

2. "POPCORN participants who reported to have suffered from (mild) COVID-19 were counted as cases" – this is an important limitation of the study that needs to

At the time the control participants were recruited, there was only very limited availability of COVID-19 tests. This limitation is now added to the discussion.

3. “Participants in all cohorts had to be at least 18 years of age, and SARS-CoV-2 cases were either confirmed by PCR or CT-scan (COVID-19 Reporting and Data System (CO-RADS) with a score of 4-5) or were suspected cases, as there was limited testing capacity at the start of the pandemic.” Here, a possible bias is observed, as some cases were not confirmed. It would be relevant to inform the percentage of those considered suspect cases, but without laboratory confirmation. Should only suspect cases be excluded?

We do not have information on how each participants’ diagnosis was made. However, we are fairly certain that the number of misclassified positive patients is very low. At the time of the start of the pandemic, patients were classified as positive/suspected if a subsequent test was deemed non-discriminatory, e.g. when they presented with complaints of COVID-19 and they were exposed to family members with the disease. Importantly, the patients included in the cohorts early in the pandemic were hospitalized and admitted wards or intensive care units because of very severe COVID-19. Due to strain on the Dutch healthcare system at that time, there was no capacity to admit less severe patients, to ICUs for example (Brinkman et al. Strain on Scarce Intensive Care Beds Drives Reduced Patient Volumes, Patient Selection, and Worse Outcome: A National Cohort Study Critical Care Medicine 2024.). In addition, during the beginning of the pandemic, severe respiratory complaints that required admission, with the typical CORADS CT, was a definite diagnosis in the Netherlands (Termorshuizen et al. Characteristics and outcome of COVID-19 patients admitted to the ICU: a nationwide cohort study on the comparison between the consecutive stages of the COVID-19 pandemic in the Netherlands, an update Ann Inten Care 2024). Later, with PCR capacity surge, PCR became standard of care, reducing the need for the CORADS CT diagnostic system, and this was also in each of our patients. So, to our opinion, the question raised, although important to address from a methodological point of view, is merely a theoretical one, when considering the dynamics of clinical practice during the beginning and later phases of the pandemic.

4. “Former COVID-19 patients and non-COVID-19 controls were sent one or more questionnaires (depending on the cohort) on symptoms associated with post-COVID For peer review only condition” – but was there no standardization? One or more questionnaires?

Yes, there was standardization, but depending on (startdate) of the cohort participants received at least one and sometimes more than one questionnaire.

5. “CORFU participants were divided into four subgroups: 1) participants without a known SARS- CoV-2 infection, i.e., the controls, 2) participants with proven SARS-

CoV-2 infection without hospitalization" – rewrite this differently, as suspect cases cannot be considered confirmed cases.

We have changed this to: participants with proven or suspected SARS-CoV-2 infection without hospitalization

6. "...4) participants with SARS-CoV-2 infection with intensive care unit (ICU)" – was there no analysis of a subgroup with mechanical ventilation, or with hemodynamic dysfunction, or with any other severity criterion?

This would have been an interesting addition to our analyses, but unfortunately, we don't have this information available.

7. "SARS-CoV-2 infection with intensive care unit (ICU) admission. Participants of the cohorts were invited to complete the CORFU questionnaire at 3, 6, 12, 18, and 24 months after initial SARS-CoV-2 infection" – it is necessary to specify how the invitation to respond to the questionnaire was made. Contact via email? In person during an outpatient visit?

The CORFU questionnaire was digitally administered at 3, 6, 12, 18 and 24 months after COVID-19 via a web-based survey or, if requested, on paper. Participants were contacted by email.

8. "The timing of the CORFU questionnaire was determined by the participant's date of first infection (diagnosis and/or admission). Due to the timing of study initiation, some participants could not complete earlier questionnaires, depending on the cohort; most participants completed either 1 or 2 during follow-up." This is already a result, not methodology. An important possible bias, as most completed only 1 or 2 questionnaires.

We have added this to the methods section in order for the intended reader to acknowledge before delving into the results section, that this is not a study with a longitudinal design in which all participants had the same follow-up moments. This, to us, is a crucial aspect of the methods of this study, and necessary to follow the choices we made in the rest of the methods section. Furthermore, we do not see this as bias, as we refrain from longitudinal analyses.

9. "Depending on the cohort and patient preference, questionnaires were completed either digitally or on paper" – again, how were the patients contacted? By email? Another way?

Participants were contacted by email. The data flow is further explained in the protocol paper.

<u>Data Collection:</u>

1. "Headache as a symptom was added to the CORFU questionnaire after June 28, 2022, as early studies reported headache as a symptom associated with post-COVID condition." Was the percentage of questionnaires that included this variable specified?

No, this percentage was not specified.

2. "In addition, we asked for vaccination status (having had one or more versus none)." What is the relevance of this? Was it indicated when the patient received the vaccine?

The relevance of asking participants for their vaccination status, was to see whether the complaints of the participants was potentially caused or detoriated by the vaccination. Also, we would want to evaluate whether participants with an incomplete vaccination status were more prone to PCC symptoms than others with a complete vaccination status.

3. "Second, we dichotomised symptoms associated with post-COVID condition into being present or absent. A symptom was registered as 'present' when it was scored at least moderately severe (3 or above on a 5-point Likert scale)." Better specify whether symptoms were considered existent only with a score of 3 or higher. What is the reason for this criterion? Why disregard patients with milder symptoms?

We have changed our wording; in the manuscript we use existent and absent instead of present and absent. After extensive consideration among the clinicians involved in the CORFU study, we concluded that only moderately severe symptoms were relevant.

4. "We computed the percentage of patients vaccinated at the time of completing their 24- month questionnaire and computed the percentage of vaccinated and unvaccinated patients that exhibited post-COVID symptoms, stratified by the four subgroups." Check if vaccination status is included in the study objectives. It doesn't make much sense, as it does not analyze when the vaccine was administered.

*Vaccination status is described as one of the determinants in our protocol pape*r *and is therefore computed in this manuscript.*

<u>In Results:</u>

1. "Incluímos 4.291 participantes que completaram um total de 5.523 questionários (Tabela 1). Isso incluiu 3.086 (72,0%) controles não COVID." We notice a high percentage of controls with few questionnaires answered by the number of participants. This can generate bias, as only patients who persisted with symptoms may have continued to answer the questionnaires.

Indeed, for this exact reason we have described this as one of the limitations of our study.

2. "We received the most questionnaires from former COVID-19 patients on the 24-month follow-up moment, i.e., 904." It may be that there was a tendency to answer the questionnaire if the symptom was persistent.

Yes, this is indeed a pitfall. At the same time, patients with the most serious symptoms might not have been able to fill in the questionnaire due to the impact of these symptoms on their daily lives.

3. "The mean age was lower in the control group…" - Possible selection bias, as the controls are younger.

Agree, this is also described in the discussion section of this paper.

4. The male-to-female ratio was close to equality in the control group (48.7% male), but in the clinical subgroups, there were substantially more males than females, with the highest percentage of males in the ICU subgroup. This difference in gender can impact, as it is known that post-COVID symptoms are more prevalent in women.

We acknowledge the effect of gender on the prevalence of post-COVID symptoms. For that reason, we have adjusted our regression models for gender (and other potential confounding variables). After adjustment, estimates will be unaffected by gender differences between groups. Moreover, as we agree on the substantial gender-effect, we are now working on a separate paper that revolves around these differences.

5. In the control group and the subgroup of non-hospitalized patients, asthma was the most frequently reported chronic disease (11.5% and 10.9%, respectively). For ward patients, it was diabetes and arrhythmia or palpitations, followed closely by osteoarthritis and low back pain. In addition to being younger, the control group was also characterized by milder comorbidities. Another potential bias. Was this corrected? There was no matching of cases for the control group patients.

It is well known in the epidemiological literature, that matching greatly increases the risk of bias in estimates. Moreover, matching variables provide no useful information anymore after used for matching. For that reason, we used multivariable regression models to adjust for any potential confounding. Please find above a directed acyclic graph with which we determined what variables should be in the minimum adjustment set.

6. "For the ward patients, this was diabetes and arrhythmia or palpitations, followed closely by osteoarthritis and low back pain" - strange palpitations and low back pain

placed as comorbidities. Also, arrhythmia disease as a comorbidity may reveal inclusion bias of patients in a cohort that evaluated cardiac patients.

We chose to include many comorbidities in our data collection as their symptoms may also be associated with post COVID and with quality of life. These comorbid conditions were also subsequently used to adjust regression models, as they could act as confounders. One of the cohorts was indeed set up initially with research questions related to cardiac diseases in mind. However, there was no other selection criterium related to this applied to their participants. Hence, all patients with COVID-19 presenting at the participating hospitals, regardless of being cardiac patients, were eligible. Therefore, no selection bias had taken place,

7. It seems that the symptoms were judged for severity by the Likert scale. There is a need to clarify whether the presence of symptoms was defined by the scale (3 or more).

Symptoms were seen as present/existent when they were rated with at least a score of 3 on the 5-point Likert scale.

8. "Of all former COVID-19 patients, 89.0% had been vaccinated at least once at 24 months. We observed large differences in vaccination between rates across subgroups. The lowest rates were found in the non-hospitalised patient group (67.1%) and the highest in the patients admitted to the ward (95.7%). In the former ICU patients, this was 87.9% (p<0.001). At 24 months, 43.9% of vaccinated non-hospitalised patients had at least one post-COVID symptom present compared to 39.6% of non-vaccinated non-hospitalised patients (p= 0.753), 65.3% of vaccinated patients admitted to the ward had at least one symptom compared to 59.1% for non- vaccinated patients (p=0.712), and 64.1% of ICU patients had at least one symptom present compared to 56.7% for non-vaccinated ICU patients (p=0.559)" Among all former COVID-19 patients, 89.0% had been vaccinated at least once at 24 months. We observed large differences in vaccination between the rates across subgroups. The lowest rates were found in the non- hospitalised patient group (67.1%) and the highest in the patients admitted to the ward (95.7%). In the former ICU patients, this was 87.9% (p<0.001). At 24 months, 43.9% of vaccinated non-hospitalised patients had at least one post-COVID symptom present compared to 39.6% of non- vaccinated non-hospitalised patients (p=0.753), 65.3% of vaccinated patients admitted to the ward had at least one symptom compared to 59.1% for non-vaccinated patients (p=0.712), and 64.1% of ICU patients had at least one symptom present compared to 56.7% for non-vaccinated ICU patients (p=0.559)". This vaccination analysis is irrelevant as the vaccination timing was not checked whether before the acute infection or after, and at what point. It is not possible to verify any association between vaccination and post-COVID symptoms or the

severity of the acute illness, whether hospitalization was needed or not. Were the controls analyzed for vaccination?

Participants in our cohorts had their COVID-19 before vaccination. What we did not know, is when they received their vaccination relative to the questionnaires they completed on post-COVID symptoms. This is due to the fact that vaccination status was only added to the last batch of CORFU questionnaires that we sent. Therefore, we could compute differences in vaccination rates between COVID-19 severity groups, but we could not study the association between receiving vaccination(s) and post-COVID symptoms. We did not perform analyses on vaccination status and the control group.

9. "At 24 months after infection, the mean and standard deviation (SD) of self-rated health on the EQ-VAS were 71.6 (17.9) for non-hospitalized patients and 70.0 (17.3) and 71.4 (17.5) for participants admitted to the ward or ICU, respectively. This was significantly lower than the control group mean EQ-VAS at 75.6 (17.7) (p<0.001 for controls versus former COVID-19 patients)…" - It was lacking to inform if there was a difference among the COVID groups.

We have used null-hypothesis testing as much as possible only for major contrasts, so as not to put the focus too much on p-values and dichotomization of results into "significant" and "not significant". For that reason, we have presented many point estimates and (after revising according to another reviewer) 95% confidence intervals around those estimates. These confidence intervals make null-hypothesis testing possible, without focusing on p-values, but on effect size and precision.

<u>In Discussion:</u>

1. "Possible cognitive dysfunction was not related to the SARS-CoV-2 infection but post-intensive care syndrome (PICS) could have played a role in this. Also, since the ICU's were full, these patients had to stay at the general ward. Thus the distribution on disease severity we made could have led to a distorted picture." – Review this last sentence, as such distortion would not impact the increase in the prevalence of cognitive changes in ICU patients.

We changed the sentence to: "We hypothesized that the severity of the acute disease was a predictor for the presence of post-COVID symptoms at 24 months, but this was only valid for the symptom's cognitive dysfunction and severity of fatigue. However, post-intensive care syndrome (PICS) could also have played a role in this in the ICU-admitted severity group. However, we have no means to discriminate PICS from post-COVID, as its presentation can be so much alike."
In other words: patients that were eligible for ICU admission due to the severity of their complaints, could not be admitted to the ICU as a result of the lack of capacity. This could have resulted in patients that were admitted to the genera ward, were classified as "general ward" in this study, but this show PICS symptoms.

2. "Symptoms 24 months after acute infection between the former ward and ICU patients differed only slightly, although treatment in the ICU differed (patients with SARS-Cov-2 were sedated and mechanically ventilated during their ICU admission)." Is this difference not shown? Correct the text, as a patient in the ICU is not necessarily subjected to mechanical ventilation.

In our cohorts, all COVID- patients were mechanically ventilated during their stay at the ICU.

3. "Although we expected that the EQ-5D-5L and VAS results would be lower at 24 months due to the severity during the acute phase for former ICU patients compared to patients who had not been admitted to the hospital, this was not the case in our study. The results indicate that the severity of the acute SARS-CoV-2 infection alone doesn't not predict HRQOL at the long term." The text is confusing, as the discussion indicates that several severe patients stayed in the ward because the ICU was full. The severity cannot be measured solely by ICU admission (was any other severity score used? SOFA, SAPS, APACHE, use of mechanical ventilation, need for vasoactive drugs?).

We acknowledge that home, admission to the ward, and admission to the ICU is only a proxy for COVID-19 severity. However, there is no gold standard that exists to classify participants into severity groups. In some cases, patients may not have been admitted to the ICU as their prognosis was very severe and the choice was made for the ward so that at least family members could visit. However, these patients likely did not survive and hence, were not misclassified in this study. However, we do acknowlegde other types of misclassification may have occured due to e.g. differences between hospitals. For that reason, we kept all group labels (e.g. non-hopsitalized, hospitalized (ICU) etc.) instead of renaming into e.g. "mild" and "severe".

4. "These risk factors may contribute to further improving the definition and prediction of post- COVID condition" Correct this sentence since I understand that risk factors cannot contribute to improving the definition of a condition.

We have changed the sentence to read: "These risk factors may contribute to further understanding of post- COVID condition" to remove all ambiguity.

5. "Regarding vaccination status, patients admitted to the hospital had the highest and non- hospitalised patients with the lowest vaccination rate. Since most of the patients in our study population had a SARS-CoV-2 infection before the vaccination rounds started, we could not determine, that any causal relation between

vaccination status and long-term symptoms." I understand that the vaccination variable should not be analyzed for this reason.

We agree with the reviewer and have therefore refrained from any other analyses with vaccination status as exposure, besides the unadjusted rates among subgroups. Hence, we did not try to estimate whether there were causal relationships between vaccination status and long-term symptoms.

In Strengths and Limitations:

1. "This study has several strengths. First, the long follow-up period up until 24 months after infection with the SARS-CoV-2 virus allows for estimating long-term post-COVID symptom prevalence and severity." This statement is not entirely true, as the percentage of patients "followed up" for a long time was small. As commented earlier, some bias may have occurred. What was the follow-up like? Were they invited to answer the questionnaire by phone?

This is addressed in an earlier question.

2. "Also, by grouping several cohorts, we were able to include a diverse population of patients and controls that enabled us to provide information on diverse groups based on sex, BMI, age, and severity of disease. The control population helps to shed light on the prevalence and severity of the same symptoms that are used to diagnose post-COVID condition. However, the control group was slightly younger on average, more often highly educated, and had fewer chronic diseases." There was no matching, this greatly hurts this conclusion. Therefore, this is not a strength; it is more of a limitation.

It is well known in the epidemiological literature, that matching greatly increases the risk of bias in estimates. Moreover, matching variables provide no useful information anymore after used for matching. For that reason, we used multivariable regression models to adjust for any potential confounding. Please find above a directed acyclic graph with which we determined what variables should be in the minimum adjustment set.

3. "..This could have resulted in underestimating the number of patients with severe complaints" add: led the patient not to respond to the questionnaire.

*We have added a sentence to the discussion section in which we suggest how patients with a more severe form of post-COVID may have refrained more often from responding: "For instance, **patients who did not experience any burden in daily life or perceive any symptoms, or those with such severe post-COVID symptoms that completing questionnaires poses too much of a burden, may have refrained from responding**. This could have resulted in underestimating the number of patients with severe complaints."*

4. "...we did not have many participants with multiple questionnaires during follow-up. This would have allowed for the estimation of within-person change." Add that the percentage of the universe that responded at 24 months was very small.

Due to the nature of data collection (a relatively brief period of calendar time), patients were only sent questionnaires relative to the time since their initial infection. Therefore, the number of respondents differs between follow-up moments. However, these are not indicative of response rates.

VERSION 2 - REVIEW

**Reviewer**	**4**
**Name**	**Leite, Arnaldo**
**Affiliation**	**Federal University of Minas Gerais, Departamento de Clínica Médica**
**Date**	**12-Jun-2025**
**COI**	

Dear authors, congratulations on the article. I think the corrections and justifications were appropriate and allowed for a better understanding of the work.
